# A *GRM7* mutation associated with developmental delay reduces mGlu_7_ expression and produces neurological phenotypes

**DOI:** 10.1172/jci.insight.143324

**Published:** 2021-02-22

**Authors:** Nicole M. Fisher, Aqeela AlHashim, Aditi B. Buch, Hana Badivuku, Manar M. Samman, Kelly M. Weiss, Gabriela I. Cestero, Mark D. Does, Jerri M. Rook, Craig W. Lindsley, P. Jeffrey Conn, Rocco G. Gogliotti, Colleen M. Niswender

**Affiliations:** 1 Department of Pharmacology and; 2 Warren Center for Neuroscience Drug Discovery, Vanderbilt University, Nashville, Tennessee, USA.; 3 King Fahad Medical City, Riyadh, Saudi Arabia.; 4 Department of Biomedical Engineering, Vanderbilt University, Nashville, Tennessee, USA.; 5 Department of Chemistry and; 6 Vanderbilt Institute of Chemical Biology, Vanderbilt University, Nashville, Tennessee, USA.; 7 Vanderbilt Kennedy Center, Vanderbilt University Medical Center, Nashville, Tennessee USA.; 8 Department of Molecular Pharmacology and Neuroscience, Loyola University Chicago, Maywood, Illinois, USA.

**Keywords:** Neuroscience, G protein&ndash;coupled receptors, Neurodevelopment, Seizures

## Abstract

The metabotropic glutamate receptor 7 (mGlu_7_) is a G protein–coupled receptor that has been recently linked to neurodevelopmental disorders. This association is supported by the identification of *GRM7* variants in patients with autism spectrum disorder, attention deficit hyperactivity disorder, and severe developmental delay. One *GRM7* mutation previously reported in 2 patients results in a single amino acid change, I154T, within the mGlu_7_ ligand-binding domain. Here, we report 2 new patients with this mutation who present with severe developmental delay and epilepsy. Functional studies of the mGlu_7_-I154T mutant reveal that this substitution resulted in significant loss of mGlu_7_ protein expression in HEK293A cells and in mice. We show that this occurred posttranscriptionally at the level of protein expression and trafficking. Similar to mGlu_7_–global KO mice, mGlu_7_-I154T animals exhibited reduced motor coordination, deficits in contextual fear learning, and seizures. This provides functional evidence that a disease-associated mutation affecting the mGlu_7_ receptor was sufficient to cause neurological dysfunction in mice and further validates *GRM7* as a disease-causing gene in the human population.

## Graphical abstract

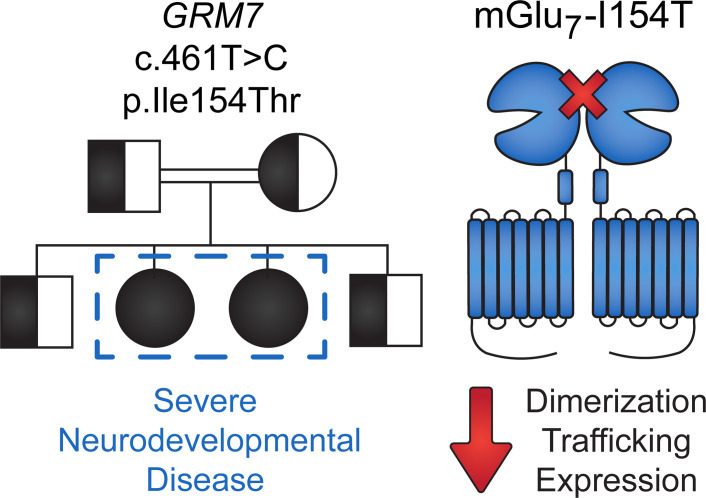

## Introduction

The metabotropic glutamate (mGlu) receptors are a class of G protein–coupled receptors that bind glutamate, the major excitatory neurotransmitter, and activate intracellular signaling pathways that modulate synaptic transmission and plasticity (1). The mGlu receptor subtype 7 (mGlu_7_) is a presynaptic G_i/o_-coupled receptor with widespread expression on glutamatergic and GABAergic neurons, and its activation leads to both constitutive and activity-dependent inhibition of neurotransmitter release (2–4). In recent years, whole exome sequencing (WES) studies have identified rare deletions and variants in *GRM7*, the human gene encoding mGlu_7_, in patients with neurodevelopmental disorders. Heterozygous deletions and point mutations have been reported in patients with autism spectrum disorder (ASD) (5–7) and attention deficit hyperactivity disorder (8), whereas homozygous point mutations have been found in rare cases of severe developmental delay, microcephaly, and epilepsy (9–11). However, the consequences of these mutations on mGlu_7_ receptor function and a causal role in disease pathogenesis have not yet been determined.

Here, we present new clinical cases of 2 sisters with severe developmental delay and epilepsy confirmed to be homozygous for a *GRM7* variant c.461T>C p.Ile154Thr (mGlu_7_-I154T). This variant was first identified by WES in 2 brothers in 2016, and detailed clinical information was recently published (9, 11). Functional studies of the mGlu_7_-I154T mutant protein in HEK293A cells and knockin mice reveal that this single amino acid change is sufficient to substantially reduce mGlu_7_ protein levels and produce a range of neurological phenotypes in mice, thus providing further support for *GRM7* as a causative gene in neurodevelopmental disorders.

## Results

We have identified a family from Saudi Arabia in which the recessive *GRM7* variant c.461T>C p.Ile154Thr segregates with severe developmental delay. Two female siblings from a consanguineous marriage presented with similar clinical features including intellectual disability, microcephaly, epilepsy, motor stereotypes, and motor impairment with early onset hypotonia that progressed into peripheral hypertonia and spasticity. Both pregnancies were uneventful with no antenatal or postnatal complications.

**Figure 1:**
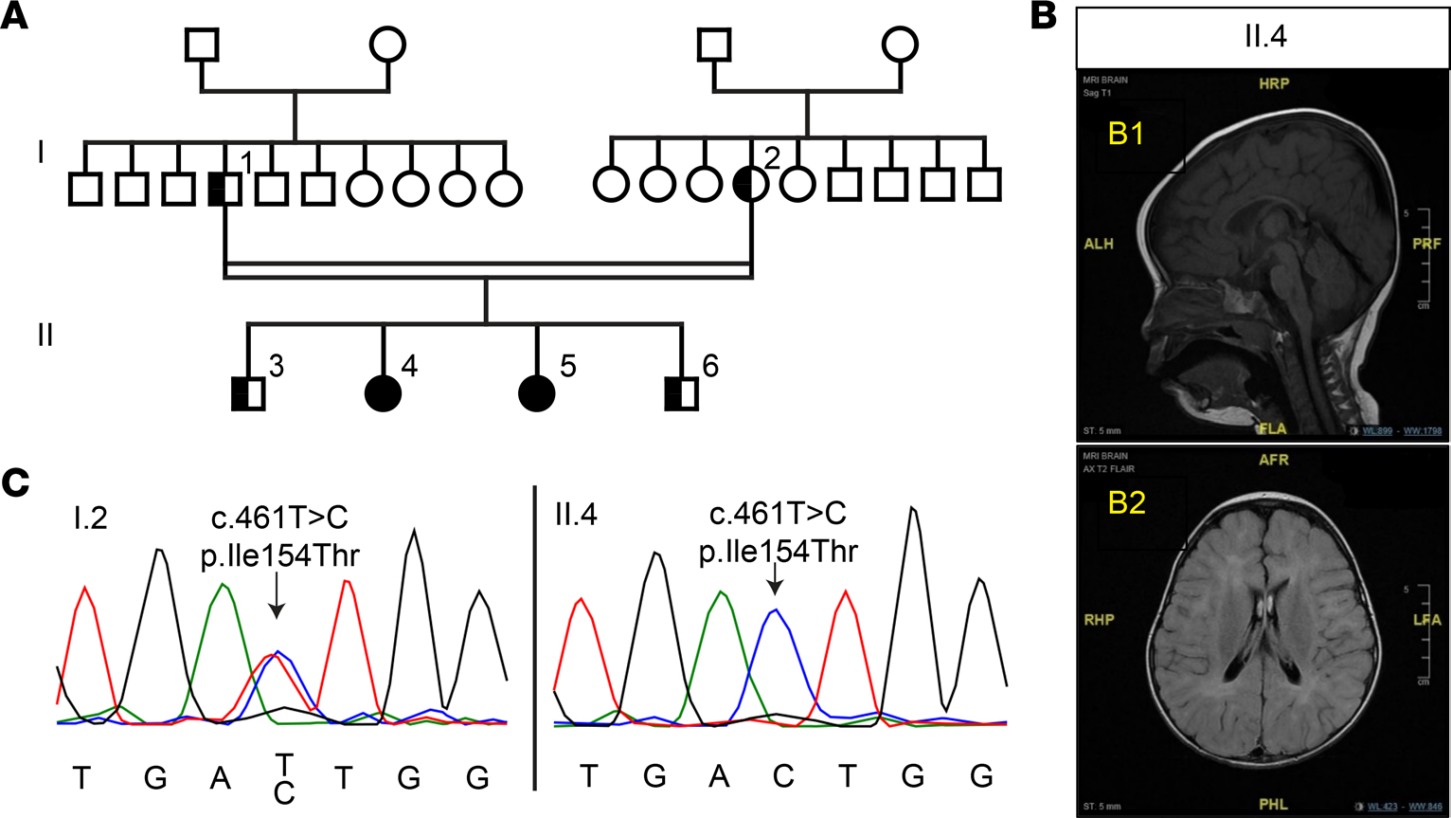
The *GRM7* c.461T>C, p.Ile154Thr variant segregates with neurodevelopmental disorder and epilepsy. (**A**) Family pedigree demonstrating affected subjects. II.4 and II.5 are proband; I.1, I.2, II.3, and II.6 are all unaffected. (**B**) MRI images of affected individual II.4. B1 is a sagittal T1 image demonstrating the finding of thin corpus callosum. B2 is an axial FLAIR image demonstrating diffuse brain atrophy with multiple patchy T2 hyperintensity in both cerebral hemispheres in subcortical and deep periventricular regions. (**C**) Sanger sequencing of the *GRM7* gene from unaffected individual I.2 showing the heterozygous variant and the affected individual II.4 showing the homozygous variant.

The eldest daughter, now age 9, (individual II.4, Figure 1A) developed hemiconvulsive focal seizures at 20 days of age, which were treated with phenobarbital for several months. Once treatment was stopped, she remained seizure free until the age of 7 years, when she developed nocturnal myoclonic seizures. These nocturnal seizures have since been controlled by levetiracetam. This patient exhibited global developmental delay from birth. She sat at 4 years and walked at 7 years of age. She is nonverbal and unable to comprehend or follow simple commands. She exhibited stereotyped clasping hand movements and head nodding at an earlier age, but these features have recently resolved.

The other daughter, now age 5, (individual II.5, Figure 1A) experienced her first seizure at 4 months of age. Her seizures were myoclonic and focal clonic in nature and have since been controlled by treatment with levetiracetam. This patient sat at the age of 2.5 years and is not yet ambulatory. She developed head nodding behavior that is similar to that of her sister. Both siblings displayed abnormal EEG, which showed frequent multifocal epileptiform discharges over the centroparietal region enhanced by sleep. MRI demonstrated diffuse brain atrophy, thin corpus callosum, and patchy T2 hyperintensities in subcortical and deep periventricular white matter consistent with hypomyelination (individual II.4, Figure 1B).

WES was performed for the eldest sibling (Figure 1A, individual II.4) and did not identify any variants in genes known to cause neurodevelopmental disorder or epilepsy at that time. A candidate variant was identified in *GRM7*, c.461T > C; p.Ile154Thr. This variant segregated appropriately after Sanger sequencing of all immediate family members: the 2 affected siblings were homozygous for this variant, whereas the remainder of the family were heterozygous carriers (Figure 1C). The heterozygous carriers were asymptomatic with no history of any neurological or psychiatric illness. This mutation has also been identified in another family in Saudi Arabia with 2 brothers exhibiting similar neurodevelopmental symptoms (9, 11), and 1 additional heterozygous case is noted in the gnomAD database in an individual of European descent (Version 3, https://gnomad.broadinstitute.org).

We next sought to characterize the functional consequence of the *GRM7* c.461T>C; p.Ile154Thr variant (hereafter referred to as mGlu_7_-I154T) to confirm that this mutation is sufficient to cause disease. Interestingly, *Grm7* knockout mice display several phenotypes that mirror those reported in the patients described in the previous paragraph. These include learning deficits, seizures, motor impairments, and stereotyped paw clasping (12–14). Based on this phenotypic overlap, we hypothesized that the mGlu_7_-I154T substitution would lead to loss of receptor function.

**Figure 2:**
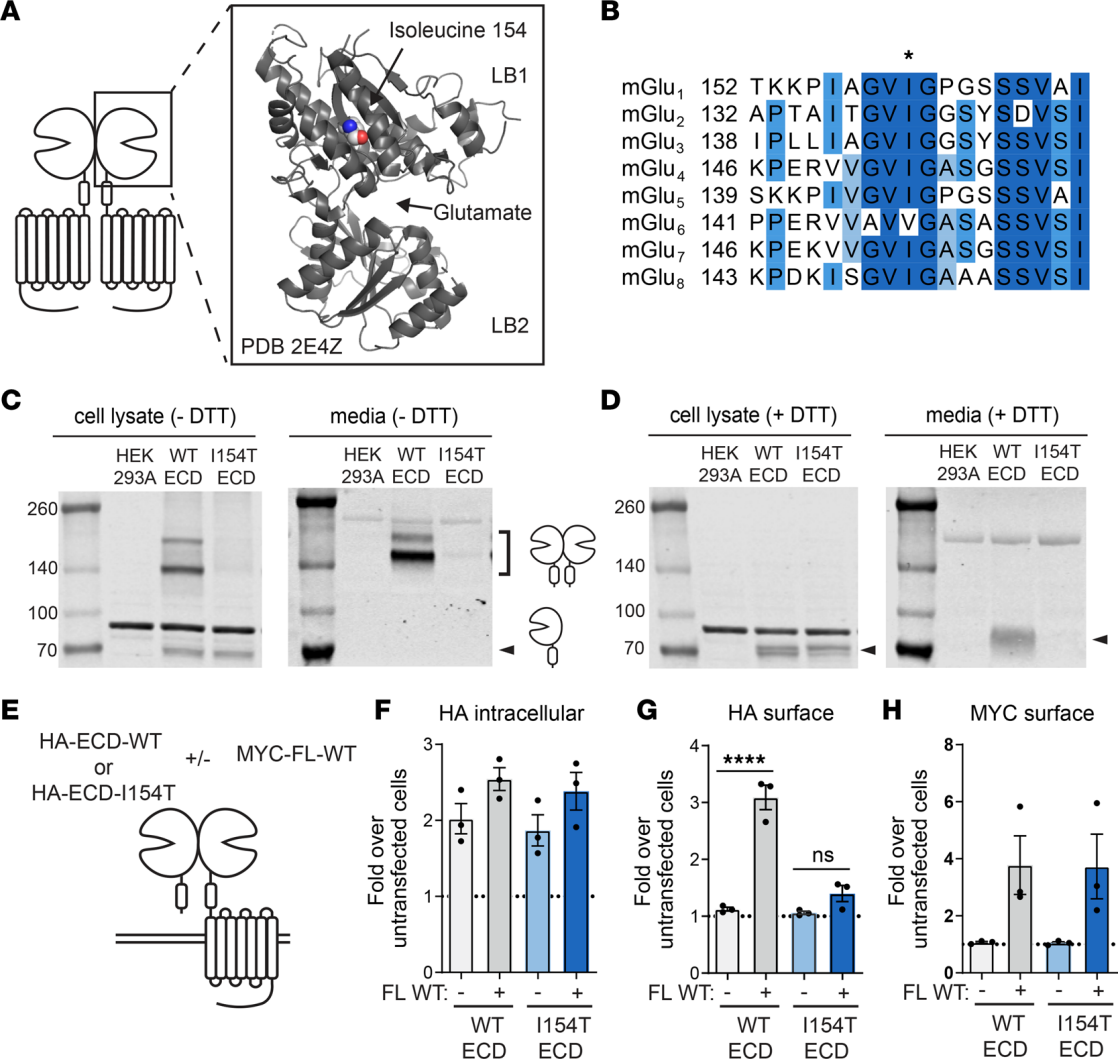
The I154T substitution disrupts the interaction of 2 ECDs within an mGlu_7_ dimer. (**A**) Location of isoleucine 154 in the structure of the mGlu_7_ ECD. PBD 2E4Z. (**B**) Protein sequence alignment of all 8 human mGlu receptor subtypes. Darker shades of blue indicate increased conservation. (**C**) Western blot of total protein lysates and media from cells expressing HA-ECD-WT and HA-ECD-I154T constructs in the absence of DTT. (**D**) Western blot of total protein lysates and media from cells expressing HA-ECD-WT and HA-ECD-I154T constructs in the presence of DTT. (**E**) Experimental design for cell surface ELISA experiments. (**F**–**H**) Quantification of (**F**) intracellular HA signal, (**G**) surface HA signal, and (**H**) surface MYC signal. *n =* 3 independent experiments with 3 technical replicates each. One-way ANOVA with Tukey’s multiple comparisons. ****P < 0.0001. ns, not significant; ECD, extracellular domain; mGlu_7_, metabotropic glutamate receptor subtype 7; FL, full-length.

The mGlu receptors are obligate dimers, and each monomer of the dimer contains a large extracellular domain (ECD), a 7-transmembrane domain, and a C-terminal cytoplasmic tail. The ligand-binding domain is within the ECD and comprises an upper lobe (LB1) and a lower lobe (LB2). This domain assumes an open-open inactive state and a closed-closed active state upon glutamate binding (15). Isoleucine 154 is positioned within a hydrophobic region of LB1, notably distant from the glutamate binding pocket (Figure 2A). When compared with the amino acid sequence of other mGlu subtypes, this isoleucine residue was highly conserved (Figure 2B). Several studies have implicated LB1 as an important interface for receptor dimerization in the inactive state (16, 17), and it has been previously shown that the ECD domains of mGlu_1_, mGlu_4_, and mGlu_5_ are secreted into media as a disulfide-bonded dimer when expressed in cell culture (18–21). To test whether the I154T mutation might disrupt receptor dimerization, we assessed the ability of a soluble ECD to dimerize upon introduction of the I154T mutation. We expressed hemagglutinin-tagged (HA-tagged) ECDs (HA-ECD-WT and HA-ECD-I154T) in HEK293A cells and collected both whole cell lysate and culture media. In the absence of a reducing agent, HA-ECD-WT from cell lysate appeared as both a dimer and a monomer by Western blot but was present in only a dimeric form in the culture media (Figure 2C). HA-ECD-I154T appeared as a monomer in cell lysate but was barely detectable as a dimer in either cell lysate or media (Figure 2C). Dimer bands were fully reduced to a monomeric form upon addition of DTT (Figure 2D). These results suggest that I154T disrupted the covalent dimerization, trafficking, and/or secretion of the mGlu_7_ ECD.

We next tested whether HA-ECD-I154T could be trafficked to the cell surface through dimerization with a full-length WT receptor. Previous studies have demonstrated that the soluble mGlu ECD can be retained on the cell surface only when coexpressed with a full-length receptor, and this has been utilized as a measure of mGlu dimerization (21, 22). To further test the effect of I154T on ECD dimerization and trafficking, we expressed HA-ECD-WT and HA-ECD-I154T constructs either alone or in combination with a full-length MYC-mGlu_7_-WT protein and measured surface expression of the N-terminal epitope tag by cell surface ELISA (Figure 2E). When expressed alone, HA-ECD-WT or HA-ECD-I154T had comparable intracellular expression (Figure 2F) but were not detectable on the cell surface (Figure 2G, light grey and light blue bars). When expressed with the full-length MYC-mGlu_7_-WT construct, we detected a significant increase in HA surface staining for HA-ECD-WT cells but not for HA-ECD-I154T cells (Figure 2G, dark grey and dark blue bars). Surface expression of the full-length MYC-mGlu_7_-WT remained constant in these experiments (Figure 2H). These results further confirm that I154T disrupts receptor dimerization and trafficking.

**Figure 3:**
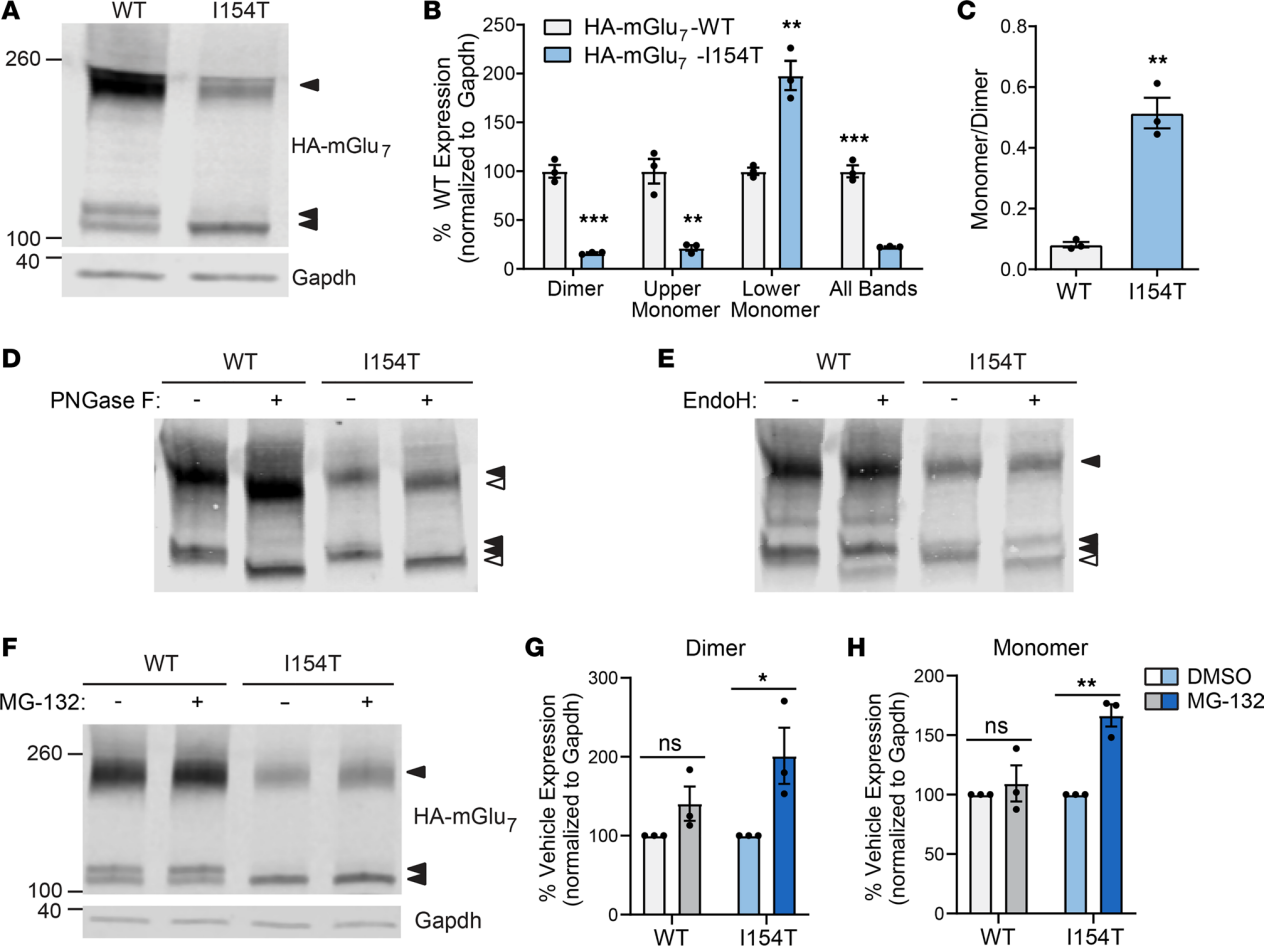
HA-mGlu_7_-I154T receptors exhibit reduced surface trafficking in HEK293A cells and are degraded by the proteasome. (**A**) Western blot of total protein lysate from HEK293A cells expressing HA-mGlu_7_-WT or HA-mGlu_7_-I154T. (**B**) Quantification of different immunoreactive bands representing mGlu_7_. (**C**) Quantification of dimer-to-monomer ratio. For **B** and **C**, *n =* 3 biological replicates. Two-tailed Student’s *t* tests. **P < 0.01, ***P < 0.001. (**D**) Western blot in the presence or absence of PNGase F treatment. (**E**) Western blot in the presence or absence of EndoH treatment. For panels **D** and **E**, open arrows indicate bands resulting from deglycosylation. *n =* 3 independent experiments. (**F**) Western blot of cell lysates after a 4-hour treatment with 10 μM MG-132 or vehicle (DMSO). (**G** and **H**) Quantification of mGlu_7_ dimer (**G**) and monomer (**H**) bands after MG-132 treatment. *n =* 3 independent experiments. Two-way ANOVA with Sidak’s multiple comparisons. *P < 0.05, **P < 0.01. ns, not significant; mGlu_7_, metabotropic glutamate receptor subtype 7.

When expressed as a full-length receptor in HEK293A cells, HA-mGlu_7_-I154T exhibited a striking reduction in total protein expression quantified by Western blot, along with altered banding patterns (Figure 3A). In the presence of DTT, HA-mGlu_7_-WT appeared primarily as a dimeric band with 2 additional monomeric bands. Expression levels of the dimer and upper monomer band were significantly reduced in cells transfected with HA-mGlu_7_-I154T, whereas expression of the lower monomer band was increased (Figure 3B). The monomer-to-dimer ratio in these blots was significantly increased for HA-mGlu_7_-I154T (Figure 3C), consistent with weakened dimerization. Park et al. recently showed that the lower monomer band observed in heterologous cells represents an immature form of mGlu_7_ that is sensitive to endoglycosidase H (EndoH), whereas the upper monomer and dimer bands represent forms of mGlu_7_ with mature glycosylation that are resistant to EndoH but sensitive to peptide: N-glycosidase F (PNGaseF, ref. 23). To confirm this in our hands, we treated cell lysates with PNGase F and EndoH. As expected, PNGase F, which removes almost all N-linked glycosylation, reduced the size of all mGlu_7_ bands (Figure 3D) and only the lower monomer band was sensitive to EndoH in both WT and I154T cells (Figure 3E). Because this band represents an immature form of mGlu_7_ present in the ER or early Golgi apparatus and mGlu receptors are known to dimerize in the ER (19), we hypothesized that I154T receptors are degraded by the proteasome through ER-associated degradation (24). Consistent with this hypothesis, treatment with the proteasome inhibitor MG-132 (10 μM) for 4 hours led to a significant increase in the expression of HA-mGlu_7_-I154T dimer and monomer bands while having no significant effect on HA-mGlu_7_-WT expression (Figure 3, F–H). Immunostaining and confocal imaging of transfected HEK293A cells showed robust expression of HA-mGlu_7_-WT throughout the entire cell, whereas HA-mGlu_7_-I154T displayed more restricted expression (Supplemental Figure 1; supplemental material available online with this article; https://doi.org/10.1172/jci.insight.143324DS1), providing further support for reduced receptor trafficking.

**Figure 4:**
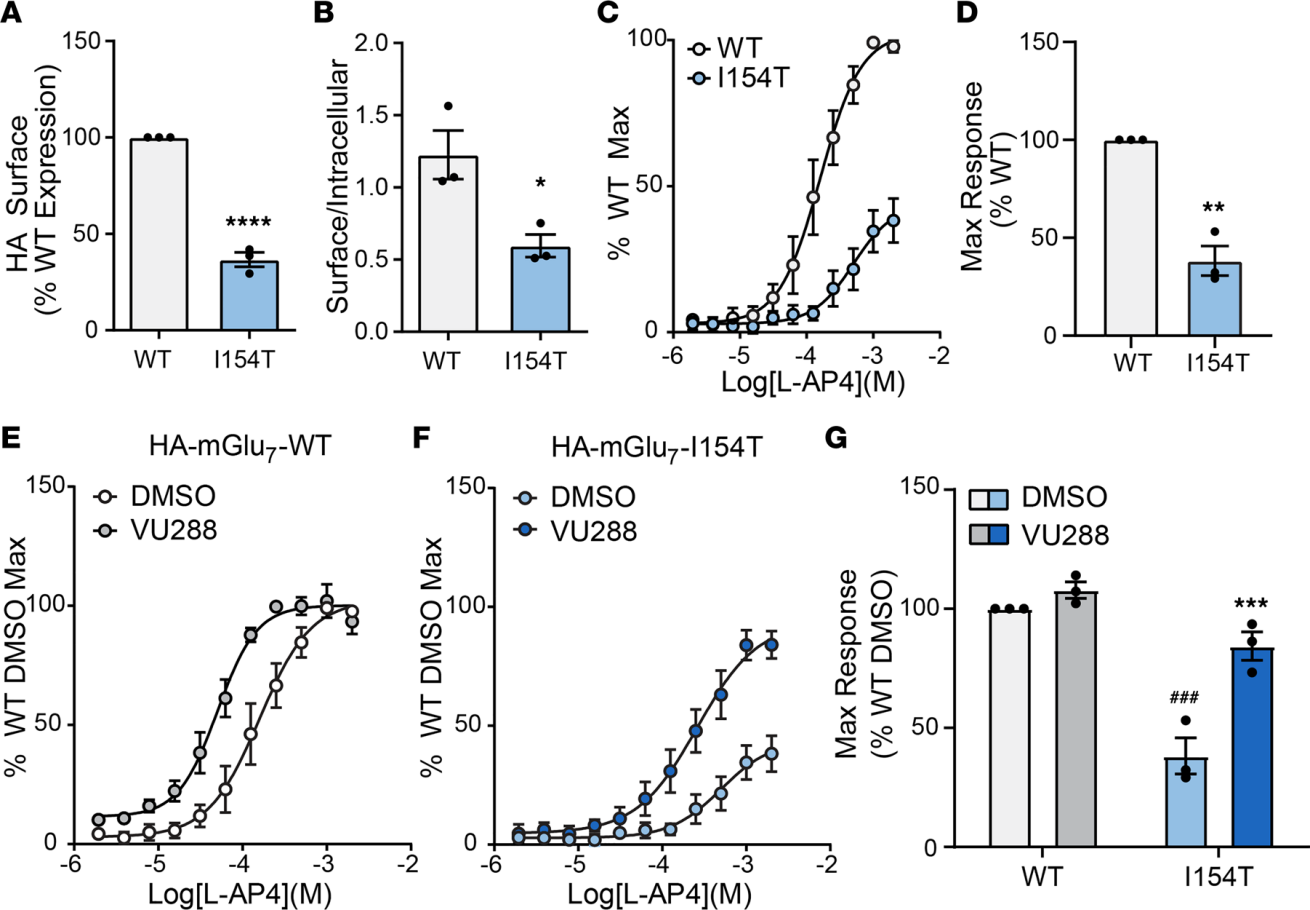
mGlu_7_-I154T receptors are expressed on the cell surface and responsive to pharmacological stimulation. (**A**) Quantification of surface HA signal in HEK293A cells expressing HA-mGlu_7_-WT or HA-mGlu_7_-I154T as measured by cell surface ELISA. (**B**) Quantification of surface-to-intracellular ratio for HA signal in cell surface ELISA experiments. For **A** and **B**, *n =* 3 individual experiments with 3 technical replicates each. Two-tailed Student’s *t* tests. *P < 0.05, ****P < 0.0001. (**C**) Concentration-response curves of cells treated with the agonist L-AP4 in a thallium flux assay. (**D**) Quantification of the maximum response in panel **C**. Two-tailed Student’s *t* test. **P < 0.01. (**E** and **F**) Concentration-response curves of cells expressing HA-mGlu_7_-WT (**E**) or HA-mGlu_7_-I154T (**F**) in response to L-AP4 with 30 μM VU0422288 (VU288) or vehicle (DMSO) pretreatment. DMSO curves are the same data presented in panels **C** and **D**. (**G**) Quantification of maximum responses in panels **E** and **F**. Two-way ANOVA with Tukey’s multiple comparisons. Stars indicate comparisons between DMSO and VU288 conditions within genotype. Pound signs indicate comparisons of DMSO conditions between genotype. ***/^###^ *P <* 0.001. For **C** and **G**, *n =* 3 independent experiments with 2 technical replicates each. mGlu_7_, metabotropic glutamate receptor subtype 7.

We next sought to determine the level of surface expression and functionality of HA-mGlu_7_-I154T receptors. We measured cell surface expression by cell surface ELISA and found a significant reduction in surface HA staining in cells transfected with HA-mGlu_7_-I154T (Figure 4A). When normalized to permeabilized wells, HA-mGlu_7_-I154T cells also exhibited a decreased surface-to-intracellular ratio (Figure 4B). Because a population of surface HA-mGlu_7_-I154T receptors was detectable, we next tested the functionality of HA-mGlu_7_-I154T receptors using a thallium flux assay (described in ref. 25). Cells stably expressing HA-mGlu_7_-WT, along with G protein–activated inwardly rectifying potassium (GIRK) channels, exhibited a concentration-dependent response to the agonist L-AP4 (Figure 4C). HA-mGlu_7_-I154T cells responded to agonist application, but the maximum response was significantly reduced (Figure 4, C and D). We further tested the ability of a positive allosteric modulator (PAM) to potentiate these responses. Preapplication of the PAM VU0422288 led to a leftward shift of the concentration-response curve of cells expressing HA-mGlu_7_-WT (Figure 4E, WT DMSO EC_50_ = 176 μM, WT VU288 EC_50_ = 57 μM) and a significant increase in the maximum response of cells expressing HA-mGlu_7_-I154T without a significant fold shift (Figure 4, F and G, I154T DMSO EC_50_ = 540 μM, I154T VU288 EC_50 =_ 308 μM). These data demonstrate that HA-mGlu_7_-I154T receptors have the potential to become activated upon pharmacological stimulation if they are properly trafficked to the cell surface.

**Figure 5:**
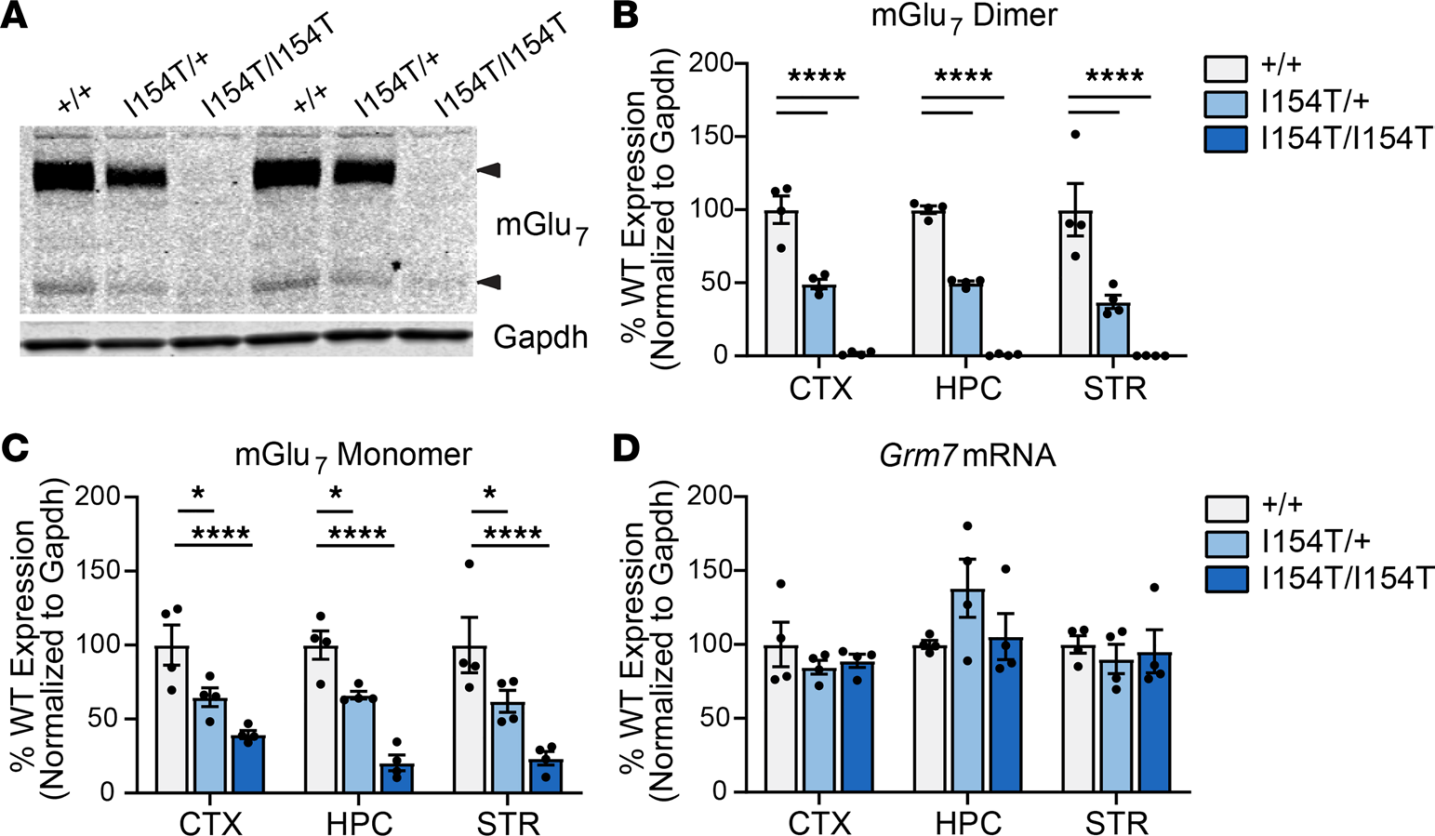
I154T-mGlu_7_ expression is reduced in vivo at the posttranscriptional level. (**A**) Western blot of total protein lysate from cortex of *Grm7^+/+^* (+/+), *Grm7^I154T/+^* (I154T/+), and *Grm7^I154T/I154T^* (I154T/I154T) mice. Arrows indicate mGlu_7_ dimer and monomer bands. (**B** and **C**) Quantification of the mGlu_7_ dimer band (**B**) and monomer band (**C**) across 3 brain regions. Two-way ANOVA with Dunnett’s comparisons to *Grm7^+/+^*. *P < 0.05, ****P < 0.0001. (**D**) *Grm7* mRNA transcript expression across each brain region. No significant difference by 2-way ANOVA. For all panels, *n =* 4 mice per genotype (2 male, 2 female). mGlu_7_, metabotropic glutamate receptor subtype 7; CTX, cortex; HPC, hippocampus; STR, striatum.

To determine the main effect of mGlu_7_-I154T in vivo, we generated a knockin mouse model using CRISPR/Cas9 technology to introduce the I154T mutation in the endogenous mouse *Grm7* gene (Supplemental Figure 2, Supplemental Table 1). Western blots of total protein lysate isolated from brain tissue of WT (*Grm7*^+/+^), heterozygous (*Grm7*^I154T/+^), and homozygous (*Grm7^I154T/I154T^*) littermates revealed an approximately 50% reduction in mGlu_7_ protein expression in *Grm7^I154T/+^* mice and little to no expression in *Grm7^I154T/I154T^* mice across 3 brain regions (Figure 5, A–C). In mouse tissue, only one monomeric band was detectable; this band was resistant to EndoH treatment in controls (Supplemental Figure 3) and was weakly detectable in *Grm7^I154T/I154T^* mice (Figure 5C). Quantification of *Grm7* mRNA transcripts from each brain region by quantitative PCR (qPCR) revealed no difference between genotypes, confirming that mGlu_7_-I154T was degraded at the posttranscriptional level in vivo (Figure 5D). Because mGlu_7_ can potentially heterodimerize with other mGlu subtypes, we also measured protein expression of mGlu_4_, a closely related Group III receptor, and mGlu_3_, a Group II receptor shown to have a high propensity to heterodimerize with mGlu_7_ (22). We found no significant difference in expression of these receptors in tissue from the cortex and hippocampus (Supplemental Figure 4).

**Figure 6:**
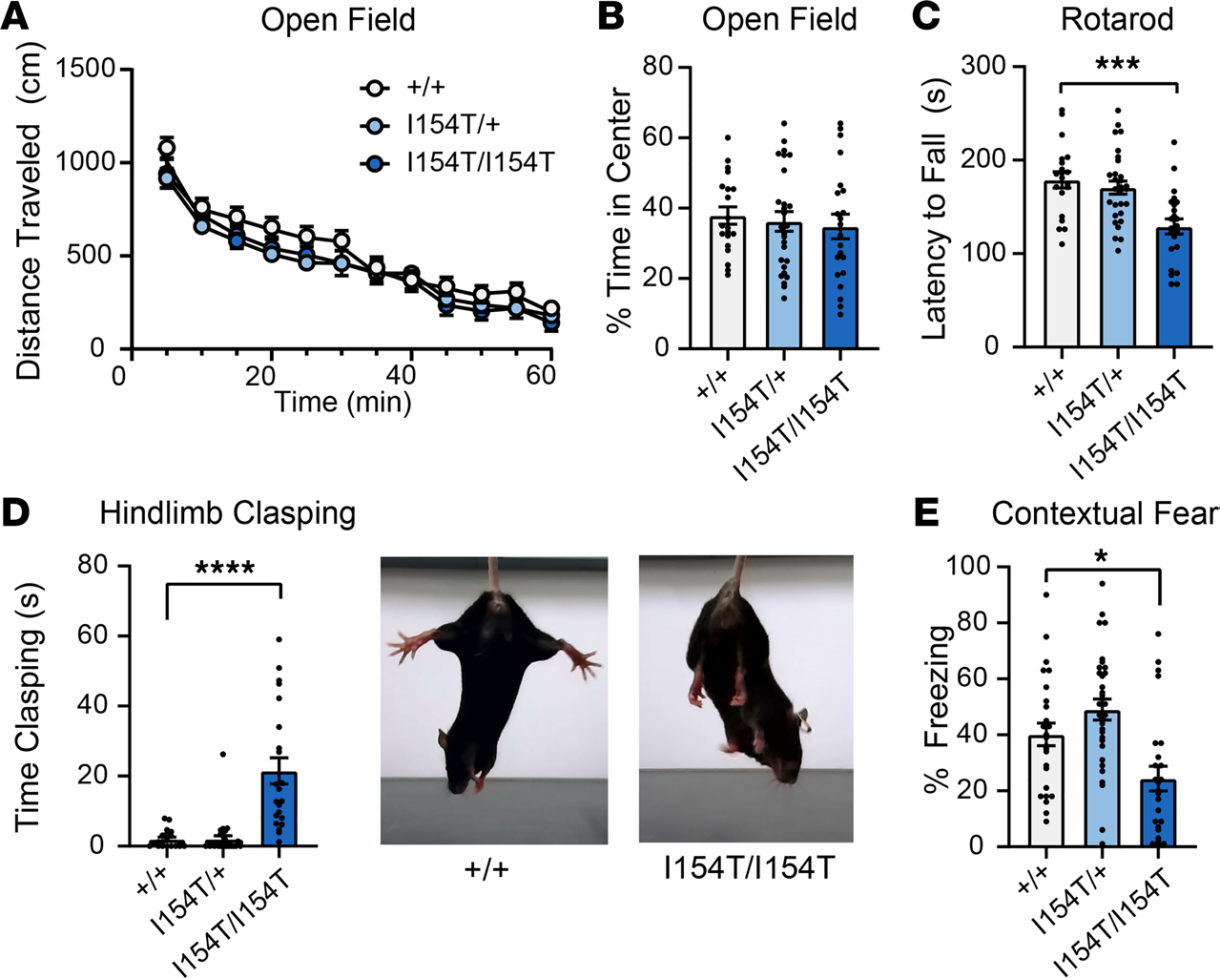
Mice homozygous for mGlu_7_-I154T exhibit deficits in motor coordination and associative learning. (**A**) Spontaneous activity over time in an open field. Genotypes are as follows: *Grm7^+/+^* (+/+), *Grm7^I154T/+^* (I154T/+), and *Grm7^I154T/I154T^* (I154T/I154T). Two-way ANOVA. (**B**) Quantification of time spent in the center of the open field. For **A** and **B**, *n =* 20 +/+, 27 I154T/+, 22 I154T/I154T. (**C**) Latency to fall from an accelerating rotarod. *n =* 20 +/+, 29 I154T/+, 23 I154T/I154T. (**D**) Quantification of hindlimb clasping. *n =* 20 +/+, 29 I154T/+, 22 I154T/I154T. Representative images shown at right. (**E**) Percent freezing in a contextual fear assay 24 hours after training. *n =* 26 +/+, 32 I154T/+, 25 I154T/I154T. For **B**–**E**, 1-way ANOVA with Dunnett’s comparisons to *Grm7^+/+^*. *P < 0.05, ***P < 0.001, ****P < 0.0001. mGlu_7_, metabotropic glutamate receptor subtype 7.

We next evaluated mGlu_7_-I154T knockin mice for phenotypes analogous to the clinical presentation of the patients described above and consistent with those reported in *Grm7* knockout animals. We found that *Grm7^I154T/I154T^* mice exhibited a small but significant reduction in body weight (Supplemental Figure 5). Spontaneous locomotion in an open field test was not different between genotypes (Figure 6A), and time spent in the center of the open field (an indicator of anxiety-like behavior) was also unchanged (Figure 6B). Despite no gross changes in locomotion, *Grm7^I154T/I154T^* mice exhibited decreased performance on the accelerating rotarod task (Figure 6C) and developed a limb clasping phenotype (Figure 6D). We also tested associative memory by contextual fear conditioning and found that *Grm7^I154T/I154T^* mice exhibited significantly decreased freezing when reexposed to the conditioning context 24 hours after training (Figure 6E). General health was assessed weekly until 20 weeks of age, and during this routine handling, convulsive seizures were observed in 8/23 *Grm7^I154T/I154T^* mice and never observed in littermates. These seizures were similar in nature to what we previously reported in *Grm7* knockout mice (12).

**Figure 7:**
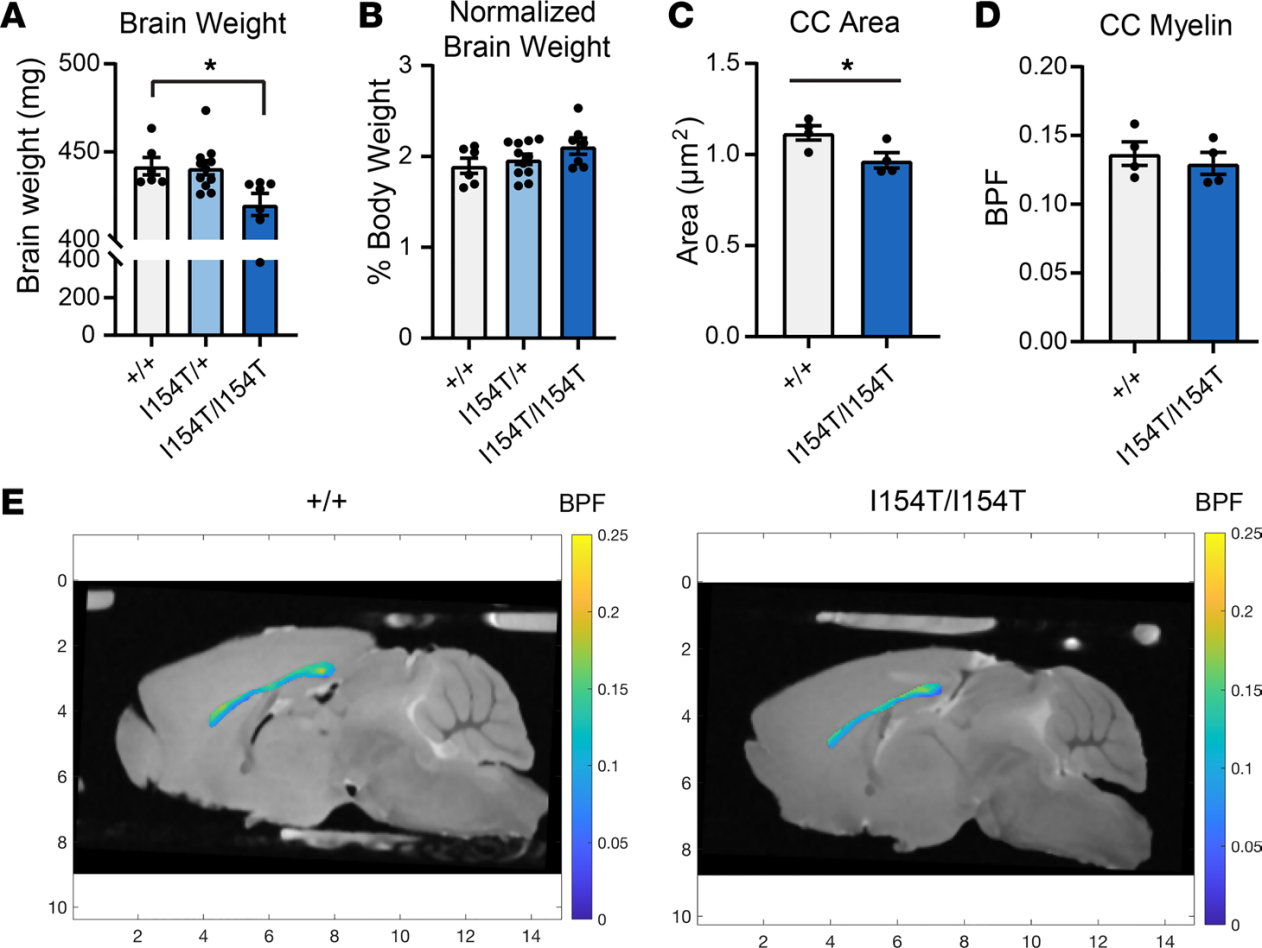
Mice homozygous for *Grm7*-I154T exhibit decreased brain weight and area of the corpus callosum. (**A**) Brain weight measured from adult mice. Genotypes are as follows: *Grm7^+/+^* (+/+), *Grm7^I154T/+^* (I154T/+), and *Grm7^I154T/I154T^* (I154T/I154T). (**B**) Brain weight expressed as percent body weight. For **A** and **B**, *n =* 6 +/+, 11 I154T/+, 7 I154T/I154T. Data analyzed by 1-way ANOVA with Dunnett’s multiple comparisons to *Grm7^+/+^*. *P < 0.05. (**C**) Area of the corpus callosum (CC) from MRI experiments. (**D**) Quantification of myelin bound pool fraction (BPF) within the CC. (**E**) Representative images showing BPF intensity within the CC. The markings on the *x* and *y* axes indicate distance in millimeters. For **D** and **E**, *n =* 4 +/+, 4 I154T/I154T. Data analyzed by 2-tailed Student’s *t* test. *P < 0.05.

Because atrophy and hypomyelination were observed in MRI scans from affected individuals, we sought to test whether the mGlu_7_-I154T mice recapitulated these abnormalities. Gross brain weight was significantly reduced in *Grm7^I154T/I154T^* mice relative to their littermates (Figure 7A); however, this difference was insignificant when brain weight was normalized to body weight (Figure 7B). We next conducted MRI scans on fixed brains to more closely examine the area and myelin content of the corpus callosum. The area of the corpus callosum was significantly reduced in *Grm7^I154T/I154T^* mice (Figure 7C). Myelin content of the corpus callosum was quantified as bound pool fraction (BPF, described in ref. 26). There was no significant difference in this measure between *Grm7^+/+^* and *Grm7^I154T/I154T^* mice (Figure 7, D and E).

## Discussion

Here we present clinical findings in parallel with the functional characterization of a disease-associated mutation affecting the mGlu_7_ receptor. Recently, biallelic mutations in *GRM7* were reported in 11 patients from 6 unrelated families, 2 of whom have the same mutation described here (11). All affected children were reported to exhibit global developmental delay, intellectual disability, epilepsy, and microcephaly. The phenotype of the individuals presented here matches the other published cases (9, 11). Their seizures have responded well to antiepileptic drugs, which is similar to patients with the same variant, but contrasts with patients with other variants who exhibited drug-resistant epilepsy (11). We also noted transient stereotypic movements in these patients in the form of head nodding and hand clasping. Stereotypic hand clasping is well recognized as a hallmark of the neurodevelopmental disorder Rett syndrome (RTT). Notably, we have proposed mGlu_7_ as a target for RTT due to decreased mGlu_7_ expression in RTT autopsy tissue and mGlu_7_’s role in promoting synaptic plasticity (27). Activation of mGlu_7_ can regulate release of glutamate and GABA, both of which have been shown to contribute to RTT-related phenotypes (28–30). Taken together with the fact that *Grm7* knockout mice recapitulate phenotypes often observed in neurodevelopmental disorders (12), it was likely that *GRM7* clinical variants would lead to loss-of-function of the mGlu_7_ receptor. However, this hypothesis had not been directly tested, leading us to determine the functional consequence of the mGlu_7_-I154T mutation.

Our data demonstrate that the I154T substitution reduced receptor dimerization, impaired cell surface trafficking, and ultimately led to a substantial decrease in total receptor expression. Isoleucine 154 is situated in the core of the LB1 domain, which harbors residues that are important for resting state dimerization of mGlu receptors (16, 17). mGlus are also known to dimerize in the ER (19), resulting in the possibility that weakened dimerization and/or stability may lead to ER retention of mGlu_7_-I154T. Interestingly, mutations affecting the N-terminal domain of mGlu_6_ have been shown to accumulate in the ER (31). In support of this hypothesis, we observed an accumulation of an EndoH-sensitive band in HEK293A cells expressing HA-mGlu_7_-I154T, representing an early glycosylated form of the receptor. We also observed an increase in mGlu_7_-I154T expression in cells treated with a proteasome inhibitor but no significant effect on mGlu_7_-WT receptors with our treatment conditions. These findings suggest that mGlu_7_-I154T receptors are degraded in part by the proteasome, which is the final step of the ER-associated degradation pathway. It should be noted that degradation of mGlu_7_ by the proteasome has also been shown to occur after agonist-induced receptor internalization, a distinct process from ER-associated degradation (32), and that fully deglycosylated mGlu_7_ is degraded by the autophagolysosomal pathway (23). The mechanisms that regulate ER export and early trafficking of mGlu_7_ are currently poorly understood, and the relative importance of dimerization and N-glycosylation remain to be determined.

In mice homozygous for the I154T mutation, mGlu_7_ protein expression was nearly absent, suggesting that mGlu_7_ is more tightly regulated in neurons compared with HEK293A cells. We observed no change in *Grm7* mRNA transcript levels in brain tissue, confirming that the mGlu_7_-I154T receptor is degraded at the posttranscriptional level in vivo. Altogether, these data demonstrate that mGlu_7_ protein expression was highly regulated by quality-control mechanisms that recognize and degrade mGlu_7_-I154T receptors, and that the ECD played a critical role in protein expression and trafficking. Consistent with an almost complete lack of mGlu_7_ protein expression, *Grm7^I154T/I154T^* mice exhibit similar phenotypes as those reported in global *Grm7* knockout mice, including deficits in motor coordination, impaired contextual fear memory, and seizures (12–14). Importantly, these phenotypes parallel the clinical description of patients that are homozygous for this variant.

Despite having about 50% protein expression, heterozygous *Grm7^I154T/+^* animals appear phenotypically normal, similar to our previous finding that *Grm7^+/-^* mice were not different from their WT littermates (12). The lack of phenotype in *Grm7^I154T/+^* mice suggests that I154T receptors do not have a significant dominant negative effect on WT mGlu_7_ receptors or other mGlu receptor subtypes in vivo. We predict that in the heterozygous state, I154T receptors are degraded early in biosynthesis and that essentially all expressed mGlu_7_ protein is WT; however, we did not directly measure this. The heterozygous carriers in the family presented here were asymptomatic; however, heterozygous deletions in *GRM7* have been reported in patients with ASD and ADHD (6, 8). Therefore, heterozygous *GRM7* mutations may be associated with disease only in the presence of other genetic or environmental factors. Altogether, the clinical, biochemical and mouse model data presented here establish that the recessive mGlu_7_-I154T variant can cause a neurodevelopmental syndrome in humans by essentially producing the same effect as gene deletion. Future studies, both clinical and preclinical, should address how modest reductions in mGlu_7_ expression may increase the risk of disorders such as ASD and ADHD.

In future studies, it will also be important to test whether other clinical *GRM7* mutations lead to loss-of-function and delineate whether this loss is due to reduced receptor expression or function. If some variants lead to altered function with retained expression, there may be an opportunity for therapeutic intervention by treatment with compounds that modulate the activity of mGlu_7_, such as positive allosteric modulators. In the case of mGlu_7_-I154T, it appears that a therapeutic strategy would need to be aimed at increasing receptor stability, trafficking, and/or expression due to the extremely low expression observed in our mouse model. In order to design such compounds, a better understanding of the mechanisms that regulate mGlu_7_ trafficking and expression is needed. The retained function of I154T receptors in heterologous cells (Figure 4) provides proof-of-concept that this approach could be successful.

Another interesting outcome from this work is an association between mGlu_7_ and myelination. Patients with homozygous *GRM7* variants exhibited characteristic neuroimaging features of cerebral atrophy and a thin corpus callosum, the latter of which reflects a paucity of white matter and a hypomyelinated state (Figure 1B and ref. 11). This consistent finding of brain hypomyelination is evidence that mGlu_7_ signaling may play a role in myelin formation and/or maintenance. We found that mGlu_7_-I154T mice exhibited reduced brain weight and a smaller corpus callosum, albeit these reductions correlated with reduced body weight. Therefore, these mice appear to have a very mild phenotype in terms of microcephaly and myelination compared with human patients. The presence of ionotropic and mGlu receptors on oligodendrocytes and their precursor cells has been established in previous studies, and it is known that glutamatergic signaling can contribute to excitotoxicity of these cells under pathological conditions (33, 34). Activation of neuronal mGlu_7_ would be expected to mitigate excessive glutamate release and may protect oligodendroctyes from excitotoxicity; however, this hypothesis remains to be tested. Additionally, mGlu_7_ has been shown to be expressed in both oligodendrocytes and precursor cells in mouse neocortex (22, 35). The role of mGlu_7_ in these cell types during development will be an interesting topic for future studies.

In summary, the clinical and functional data presented here demonstrate that a homozygous point mutation in the mGlu_7_ receptor is sufficient to cause disease phenotypes through a loss of receptor expression. Therefore, loss-of-expression or loss-of-function mutations in *GRM7* should be considered as a potential underlying cause for patients with unexplained developmental delay and epilepsy.

## Methods

### WES

WES was performed for the index case (individual II.4, Figure 1A). DNA libraries were constructed using the Agilent SureSelect Kit (Version 5). Quality control for insert size and library representation was performed using Agilent Bioanalyzer and qPCR, respectively. Sequencing was undertaken using an Illumina HiSeq 4000 to an average depth of coverage of 75×–150× with automated adapter trimming of the fastq sequences. DNA alignment, variant identification, and quality filtering were undertaken using commercially available algorithms (XNG [DNASTAR v13.0a-d/v14.0] using default parameters). Human reference assemblies were aligned against GRCh37.p13 with variant annotation using dbNSFPv.2.9.0 and dbSNP146 databases.

Nonsynonymous homozygous/hemizygous variant (SNP/indel) selection was performed using preliminary filtering parameters including a minimum depth of coverage of 20×, pNotRef ≥ 0.9 (internal probability calculation from Mapping and Alignment Quality algorithm that the variant is not the reference), Q call (Phred base quality score) ≥ 20, and a PhyloP100way_veterbrate score ≥ 2.5. Variants were triaged by their absence from publicly available databases including dbSNP146/1000G/ESP6500. Candidates were then manually curated by having a requirement for allele and/or genotype frequency of less than 1% as compared with an in-house exome database (*n =* 2564; build version 3 2020). Variants with PhyloP100way_veterbrate scores lower than 2.5 still had a requirement to meet the allele and/or genotype frequency of less than 1% using the in-house database in order to be considered further.

All variant sites were manually curated using SeqMan Pro (next-generation sequencing alignment visualization software) in order to confirm the presence of the variant and to minimize the risk of PCR strand bias with respect to either the forward (F) or reverse (R) strands (maximum ratio of 9:1 for either F/R or R/F). Nonsynonymous heterozygous, frameshift, and splice site donor/acceptor variants were screened using similar methodologies outlined above. Genomic, mRNA, and protein coding variant locations were obtained using Mutalyzer v2.0.22 (Human Genome Variation Society [HGVS] nomenclature version 2.0). Variants and their associated genes/biological pathways were correlated to clinical phenotypes using publicly available databases (e.g., OMIM, Pubmed, HGMD).

Comprehensive analysis of exome sequencing data was implemented to detect potentially damaging variants and candidate variants were classified and reported by board-certified geneticists following HGVS nomenclature and American College of Medical Genetics guidelines, whereas no variants were identified in other known disease genes. For the index case (individual II.4), a homozygous missense variant was identified in the *GRM7* gene: NM_000844.4(*GRM7*): c.461T>C (p.I154T), classified as damaging by SIFT, and probably damaging by PolyPhen-2, with a CADD score of 28.900, genotype quality 60, call quality 60, and a read depth of 161. This variant in the *GRM7* gene was classified as likely pathogenic in ClinVar (accession no. VCV000242895). Sanger confirmation for the other affected case and segregation analysis for family members was performed; see Figure 1.

### Cell culture, expression constructs, and transfection

HEK293A cells were cultured in growth media containing DMEM with 10% FBS, 20 mM HEPES, 1 mM sodium pyruvate, 2 mM GlutaMAX, 0.1 mM nonessential amino acids, and antibiotic/antimycotic. For Figure 2, C and D, cells were grown in OptiMEM with reduced serum (2% FBS) and cell culture media was concentrated using Amicon Ultra centrifugal filters (MilliporeSigma, 50 kDa cutoff). HA-mGlu_7_-WT was custom designed using the cDNA sequence for the human mGlu_7_a isoform (NM_000844.4) and synthesized as a gBlock fragment (Integrated DNA Technologies). The HA tag was inserted after the N-terminal signal peptide MVQLRKLLRVLTLMKFPCCVLEVLLCALAAAARG. This DNA fragment was cloned into the pIRESpuro3 expression vector (Clontech) using the EcoRI and NotI sites. For the MYC-mGlu_7_-WT construct, a second gBlock with a MYC tag substituted for the HA tag was subcloned into the HA-mGlu_7_-WT vector using the EcoRI site and an internal Bsu36I site. Similarly, a gBlock containing the c.461T > C p.Ile154Thr mutation was synthesized and subcloned using the EcoRI site and the Bsu36I site to generate HA-mGlu_7_-I154T. To generate HA-ECD-WT and HA-ECD-I154T, a gBlock containing the mutation c.1757G>A p.W586* was subcloned using the Bsu36I site and the NotI site. This particular mutation has also been identified in children with neurodevelopmental disease (10). All experiments were performed from HEK293A cells transfected with plasmid DNA using Fugene6 (3 μg DNA for about 2 × 10^6^ cells unless otherwise stated) or from polyclonal lines selected with puromycin from an initial 3 μg transfection.

### Cell surface ELISA

For Figure 2, D–H, HEK293A cells were transfected with 2 μg of HA-ECD-WT or HA-ECD-I154T alone or in addition to 1 μg MYC-mGlu_7_-WT. Twenty-four hours after transfection, cells were trypsinized and plated onto a 96-well plate coated with poly-D-lysine at a density of 100,000 cells per well. Twenty-four hours after plating, media was aspirated and cells were washed with PBS before fixation with 4% paraformaldehyde for 15 minutes. Cells were washed again with PBS and then incubated with 0.5% Triton X-100 for 10 minutes for permeabilization. For surface expression, wells continued to incubate in PBS without detergent. After permeabilization, cells were washed with PBS and incubated with Odyssey blocking buffer (LI-COR) for 1 hour at room temperature. Wells were then incubated overnight at 4°C with primary antibodies to either the HA tag (1:5000, Abcam, ab9110) or the MYC tag (1:1000, Cell Signaling Technologies, 71D10, catalog 2278) diluted in blocking buffer. Primary antibody was removed and cells were washed 3 times with Tris-buffered saline plus Tween 20 (TBST, 25 mM Tris, 150 mM NaCl, 0.05% Tween 20). Cells were then incubated for 1 hour at room temperature with fluorescent secondary antibody (800CW, 1:5000, LI-COR) and DRAQ5 (1:1000, Invitrogen) diluted in blocking buffer. Cells were washed 3 times with TBST and the plate was allowed to dry at room temperature before imaging with an Odyssey scanner. For each well, signal was quantified with Image Studio Light software. The signal from the 800 channel (HA tag or MYC tag) was normalized to the signal from the 700 channel (DRAQ5) to account for cell number.

### Protein isolation

To collect brain tissue, mice were anesthetized with isoflurane and decapitated. Brains were quickly removed and microdissected to isolate whole cortex, hippocampus, and striatum. Tissue was frozen on dry ice and stored at –80°C until processing. Cells were washed with ice-cold PBS, harvested by scraping, pelleted, and stored at –80°C. To isolate total protein, samples were resuspended in RIPA buffer (Sigma), homogenized with a hand-held motorized mortar and pestle, and allowed to incubate on ice for 30 minutes with occasional vortexing. Samples were then spun for 20 minutes at 15,000*g* at 4°C. The supernatant was saved and protein concentration was determined using a bicinchoninic acid protein assay (Pierce).

### Western blotting

Samples were prepared by combining total protein lysate (20 μg from cells, 50 μg from brain tissue) with 4× Odyssey loading dye (LI-COR) in the presence or absence of 250 mM DTT. For glycosidase experiments, whole cell lysates were treated with PNGase F (Promega) or EndoH (New England Biolabs) according to the manufacturer’s instructions. Samples were electrophoretically separated using a 4% to 20% SDS polyacrylamide gel and then transferred onto a nitrocellulose membrane (Bio-Rad). Membranes were blocked with Odyssey blocking buffer (LiCor) for 1 hour at room temperature and probed with primary antibodies against the HA tag (1:5000, Abcam, ab9110), mGlu_3_ (1:1000, Alomone, AGC-012), mGlu_4_ (1:1000, Abcam, ab53088), mGlu_7_ (1:1000, MilliporeSigma, 07-239), or Gapdh (1:5000, Thermo Fisher Scientific, MA5-15738). Membranes were washed 3 times with TBST and then incubated with goat anti-rabbit fluorescent secondary antibody (800CW, 1:5000, LI-COR) and goat anti-mouse fluorescent secondary antibody (680CW, 1:5000, LI-COR). Blots were imaged with an Odyssey scanner and fluorescence was quantified using Image Studio Light software. See supplemental material for images of full unedited blots

### Immunocytochemistry and confocal microscopy

HEK293A cells were plated in 24-well dishes on poly-D-lysine–coated round 12-mm coverslips (Corning 354086) and transfected with 500 ng of HA-mGlu_7_-WT or HA-mGlu_7_-I154T. Two days after transfection, cells were washed and fixed with 4% PFA for 15 minutes at room temperature. Fixed cells were washed with PBS and permeabilized with 0.25% Triton X-100 for 10 minutes. Cells were washed again and incubated with blocking buffer for 1 hour (1% BSA, 3% gelatin from cold-water fish skin in PBS). Cells were then incubated overnight at 4°C with primary antibody to the HA tag (1:500, 12CA5, Vanderbilt Antibody and Protein Resource) diluted in 1% BSA. Cells were then washed 3 times with PBST (PBS, 0.1% Tween 20), incubated with secondary antibody (1:5000, Alexa Fluor 647 AffiniPure Goat Anti-Mouse IgG, The Jackson Laboratory) along with Alexa Fluor 594 Phalloidin (Invitrogen, A12381) in 1% BSA for 1 hour at room temperature. Cells were washed 3 times with PBST and mounted using ProLong Gold Antifade Reagent with DAPI (Invitrogen, P36941). Z-stack images were collected on a Zeiss LSM 880 confocal microscope. Images are presented as maximum intensity projections generated with ImageJ software.

### Thallium flux assay

Polyclonal cell lines stably expressing HA-mGlu_7_-WT or HA-mGlu_7_-I154T along with GIRK1/2 channels were generated by transfecting 3 μg of each mGlu_7_ construct into an existing HEK/GIRK cell line as described in ref. 25 and selected for mGlu_7_ with puromycin. Polyclonal lines were maintained in growth media containing 1:1 DMEM:F12 with 10% FBS, 20 mM HEPES, 1 mM sodium pyruvate, 2 mM GlutaMAX, 0.1 mM nonessential amino acids, antibiotic/antimycotic, 700 μg/ml G418 (for maintaining GIRK1/2), and 600 ng/ml puromycin (for maintaining mGlu_7_). Thallium flux assays were performed according to methods described in ref. 25, with minor modifications. The day prior to the experiment, cells were plated in an amine-coated 384-well plate (Corning 356719) at a density of 15,000 cells per well in assay media containing DMEM with 10% FBS, 20 mM HEPES, 1 mM sodium pyruvate, and antibiotic/antimycotic. For dye loading, media was exchanged with Assay Buffer (HBSS containing 20 mM HEPES, pH 7.4) using an ELX405 microplate washer (BioTek), leaving 20 μL/well, followed by addition of 20 μL/well 2× FluoZin-2 AM (330 nM final) indicator dye (Life Technologies, prepared as a DMSO stock and mixed in a 1:1 ratio with pluronic acid F-127) in Assay Buffer. After a 50-minute incubation at room temperature, dye was exchanged with Assay Buffer, leaving 20 μL/well. Thallium flux was measured at room temperature using a Functional Drug Screening System 7000 (FDSS 7000, Hamamatsu). Baseline readings were taken (2 images at 1 Hz; excitation, 470 ± 20 nm; emission, 540 ± 30 nm), and VU0422288 (2X) was added in a 20 μL volume and incubated for 140 seconds before the addition of 10 μL of Thallium Buffer with the agonist L-AP4 (5X). Data were collected for an additional 2.5 minutes and analyzed using Excel (Microsoft) as previously described (25), and the concentration-response curves were fitted to a 4-parameter logistic equation to determine potency estimates using GraphPad Prism.

### RNA isolation and qRT-PCR

Tissue samples were homogenized by mortar and pestle and total RNA was prepared using Trizol Reagent (Life Technologies) in accordance with the manufacturer’s instructions. Total RNA was treated with DNAse and cleaned using the RNeasy Kit (Qiagen). Complementary DNA was synthesized using the Superscript VILO cDNA Synthesis Kit (Invitrogen). A no reverse-transcriptase control was run for each sample. We then performed qRT-PCR with PowerSYBR Green PCR master mix (Applied Biosystems) using cDNA representing 25 ng of starting RNA and primers targeting *Grm7* and *Gapdh*. Primer sequences are as follows: *Grm7* 5′-CTCGACCAGATCAACAGCGA-3′ and 5′-CAGGAGCCGTGGATGCATAA-3′, *Gapdh* 5′-AGGTCGGTGTGAACGGATTTG-3′ and 5′-GGGGTCGTTGATGGCAACA-3′. Ct values for each sample were normalized to *Gapdh* expression and analyzed using the ΔΔCt method.

### Generation of mGlu_7_-I154T knockin mice

Mice expressing the p.Ile154Thr mutation in the endogenous *Grm7* gene were generated on a C57BL/6J background by the Vanderbilt Genome Editing Resource. Two overlapping crRNAs were designed targeting the mutation site within exon 1 of *Grm7*. The crRNAs were microinjected into embryos along with CRISPR/Cas9 ribonucleoprotein complex and a 180 bp single-stranded DNA donor sequence for homology-directed repair. This donor template was designed to incorporate a single-base substitution at position 461 to create the Ile154Thr mutation (ATT to ACT), along with 2 silent mutations at Val153 (GTG to GTA) and Gly155 (GGG to GGT). The I154T mutation introduced a new BsrI restriction site, which was used for genotyping. Founder mice were screened by PCR amplification followed by BsrI digestion (New England Biolabs). See Supplemental Table 1 for sequences of crRNAs, repair template, and primers.

Founder mice positive for cleavage were further confirmed by Sanger sequencing. One male founder was bred to a female C57BL/6J mouse (The Jackson Laboratory) to confirm germline transmission. One heterozygous male offspring from the resulting litter (generation N1) was sequenced to confirm the absence of off-target editing within the region of the repair template. This male mouse was backcrossed once again to female C57BL/6J mice (The Jackson Laboratory). Heterozygous mice from the resulting generation (N2) were used as breeders to generate the colony. Littermate controls were used in all experiments.

### Animal behavior

All animals used in this study were group-housed with food and water given ad libitum and maintained on a 12-hour light/dark cycle with tests occurring during the light phase. Both male and female mice were used in phenotyping experiments. No significant sex differences were observed; therefore, data were combined. Mice underwent the following testing schedule starting at 6 to 7 weeks of age with a minimum of 3 days of time between each test: open field, rotarod, fear conditioning, and hindlimb clasping. For all tests, mice were habituated to the testing room for a minimum of 1 hour.

#### Open field.

Mice were placed in an activity chamber measuring 27 × 27 cm for 60 minutes where X, Y, and Z beam breaks were monitored by Activity Monitor software (Med Associates, Inc.). The total distance traveled was quantified by this software.

#### Rotarod.

Mice were placed on an accelerating rotarod (4 to 40 rpm over 5 minutes) and the latency to fall from the apparatus or complete 2 rotations without regaining control was recorded with a cutoff of 300 seconds.

#### Limb clasping.

Mice were suspended by the tail and video-recorded for 1 minute. Time spent clasping the hindlimbs was quantified by a blinded reviewer. The timer was started when 1 or both hindlimbs began to knuckle in, and the timer was stopped if the paws came apart at any point. Time was counted if 1 paw remained knuckled in while the other came away. Time was stopped when the mouse’s back was turned to the camera.

#### Fear conditioning.

On training day, mice were placed into an operant chamber with a shock grid (Medassociates, Inc.) in the presence of a 10% vanilla odor cue. After a 3-minute habituation period, 2 tone-shock pairings were administered consisting of a 30-second tone ending with a 1-second, 0.7-mA foot shock. Each tone-shock pairing was spaced 30 seconds apart and mice remained in the context for an additional 30 seconds after the second foot shock. On the next day, mice were tested for contextual fear memory by placing each animal back into the same chamber with a 10% vanilla odor cue for 3 minutes. Time spent freezing during a 3-minute testing period was quantified using Video Freeze software.

### MRI analysis of corpus callosum

Eight adult female mice (4 *Grm7^+/+^* and 4 *Grm7^I154T/I154T^*) were anesthetized with isoﬂurane and sacrificed via transcardial perfusion. The perfusion consisted of 1x PBS wash followed by 2.5% glutaraldehyde plus 2% paraformaldehyde (modified Karnovsky solution). After perfusion, brains were quickly removed from the skull and immersed in the fixative solution for 1 week. Brains were then washed with 1x PBS plus 0.01% sodium azide, changing wash 4–5 times over 1 week to remove excess fixative. In all cases, 1.0 mM gadolinium (Prohance; Bracco Diagnostics) was included in the perfusate, immersion, and wash solutions, resulting in relatively uniform distribution of Gd throughout the brain.

Four brains at a time were loaded into a three-dimensional (3D) printed MRI-compatible mouse brain holder (https://github.com/remmi-toolbox/sample-holders) and the void space was filled with perfluoropolyether fluid (Fomblin, Solvay Solexis, Thorofarem) to prevent tissue dehydration. MRI was performed using a 16-cm horizontal bore 7 T magnet, equipped with a Bruker console (Bruker Biospec), and a 25-mm diameter quadrature volume coil for transmission and reception.

Anatomical images were acquired using a 3D RARE imaging sequence, rare factor = 4, effective echo time = 14.4 ms, repetition time = 350 ms, field of view (FOV) = 21.6 × 16.2 × 14.4 mm^3^, 50-μm isotropic resolution, 2 averaged excitations, and scan time ≈ 4.5 hours. Quantitative magnetization transfer (qMT) imaging was performed with the same FOV and 150-μm isotropic resolution, using a selective inversion recovery sequence, similar to previous work (26, 36). Images were collected using a rare factor = 8, with N_I_ = 12 inversion delays log spaced between 5 ms and 1 second, with a fixed predelay equal to 200 ms, 2 averaged excitations, and a scan time ≈ 6 hours.

qMT analysis was performed using tools available at https://remmi-toolbox.github.io The N_I_ image magnitudes were fitted voxel-wise to the Bloch-McConnell equations describing longitudinal relaxation and magnetization transfer between free water and bound macromolecular protons (36, 37). This analysis resulted in estimates of the relative size of the free and bound proton pool sizes (M_0f_ and M_0b_, respectively) and the BPF was defined as BPF = M_0b_/(M_0b_ + M_0f_).

Each 3D anatomical image was manually rotated to present a mid-brain sagittal slice, from which a corpus callosum region of interest (ROI) was manually defined. The BPF maps were interpolated to the resolution of the anatomical images, and then using the same rotations and ROI, a mean BPF for the mid-brain corpus callosum was computed. This analysis was repeated 3 times and each time the ROIs were manually defined by a different person. Values for area and BPF were averaged for each mouse.

#### Statistics.

All data shown represent mean ± SEM. P values of less than 0.05 were considered significant. Data were analyzed by a 2-tailed Student’s *t* test or 1- or 2-way ANOVA where appropriate

#### Study approval.

All clinical data have been shared with informed consent of the patients’ family with approval from the Institutional Review Board (IRB) at King Fahad Medical City (IRB no. 16-324). All animal studies were approved by the Institutional Animal Care and Use Committee for Vanderbilt University School of Medicine. Animals were cared for in accordance with the *Guide for the Care and Use of Laboratory Animals* (National Academies Press, 2011).

## Author contributions

Research was designed by NMF, AA, RGG, and CMN. AA and MMS provided all clinical data. MRI experiments and analysis were performed by MDD, JMR, GIC, and NMF. All other experiments were performed and analyzed by NMF, ABB, HB, and KMW. The manuscript was written by NMF, AA, and CMN with input from the other authors. Funding and resources were provided by CWL, PJC, and CMN.

## Supplementary Material

Supplemental data

## Version 1 01/21/2021

In-Press Preview

## Version 2 02/22/2021

Electronic publication
