# Supplementary material for: A GRM7 mutation associated with developmental delay reduces mGlu7 expression and produces neurological phenotypes
Source: JCI Insight. 2021 Feb 22;6(4):e143324. doi: 10.1172/jci.insight.143324 (PMC7934925; doi:10.1172/jci.insight.143324)

Supplemental Figures and Table for:

A *GRM7* mutation associated with developmental delay reduces $mGlu_7$ expression and produces neurological phenotypes

Authors: Nicole M. Fisher, Aqeela AlHashim, Aditi B. Buch, Hana Badivuku, Manar M. Samman, Kelly M. Weiss, Gabriela I. Cestero G, Mark D. Does, Jerri M. Rook, Craig W. Lindsley, P. Jeffrey Conn, Rocco G. Gogliotti, Colleen M. Niswender

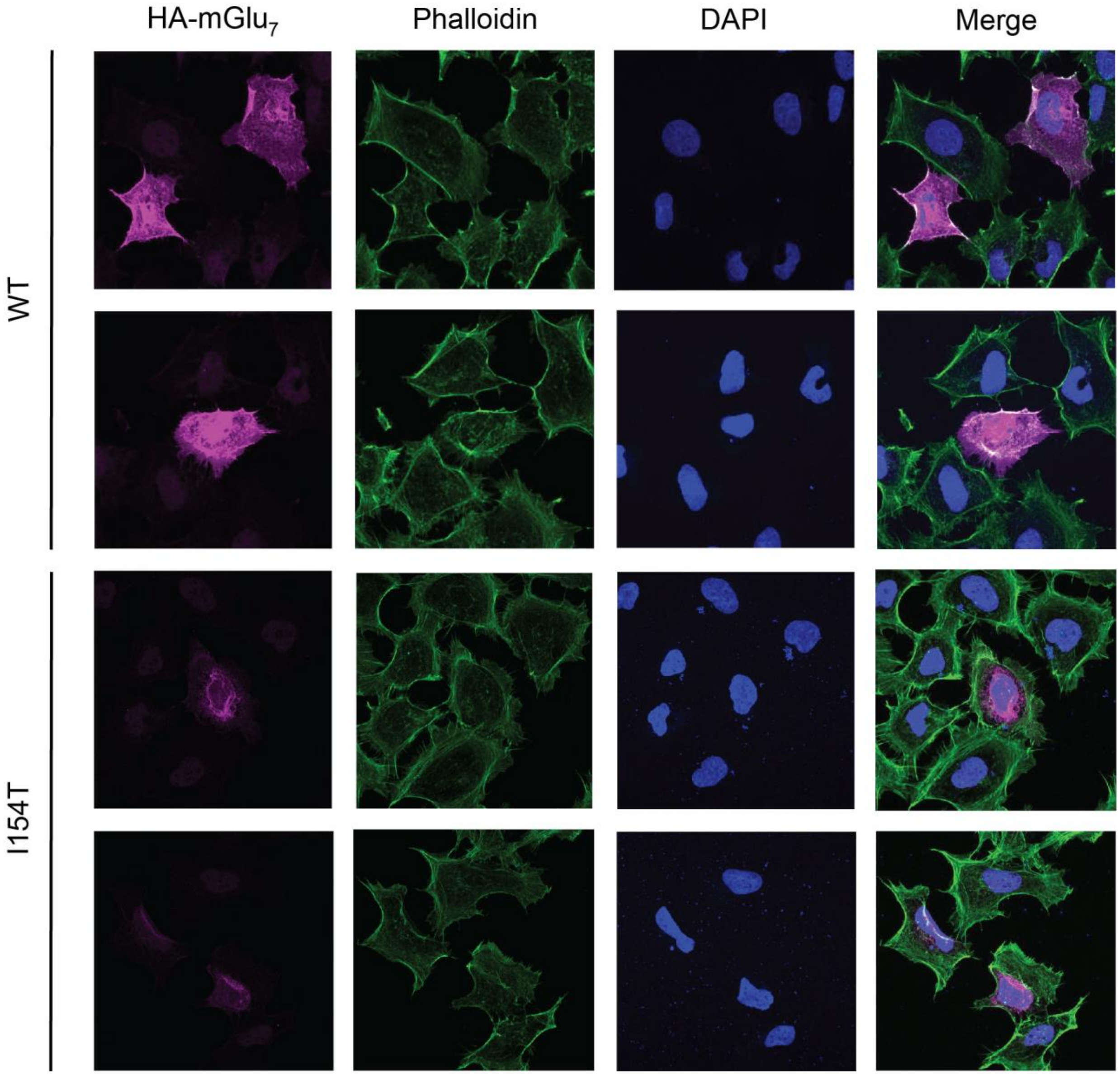

Figure S1. HA-mGlu7-WT exhibits higher, more widespread expression relative to HA-mGlu7-I154T.
Maximum intensity projections of HEK293A cells transfected with HA-mGlu7-WT or HA-mGlu7-I154T (magenta). Phallodin (green) was used to stain actin filaments to visualize the rest of the cell and nuclei were stained with DAPI (blue). Representative images from N = 3 independent experiments. Images taken at 63X magnification.

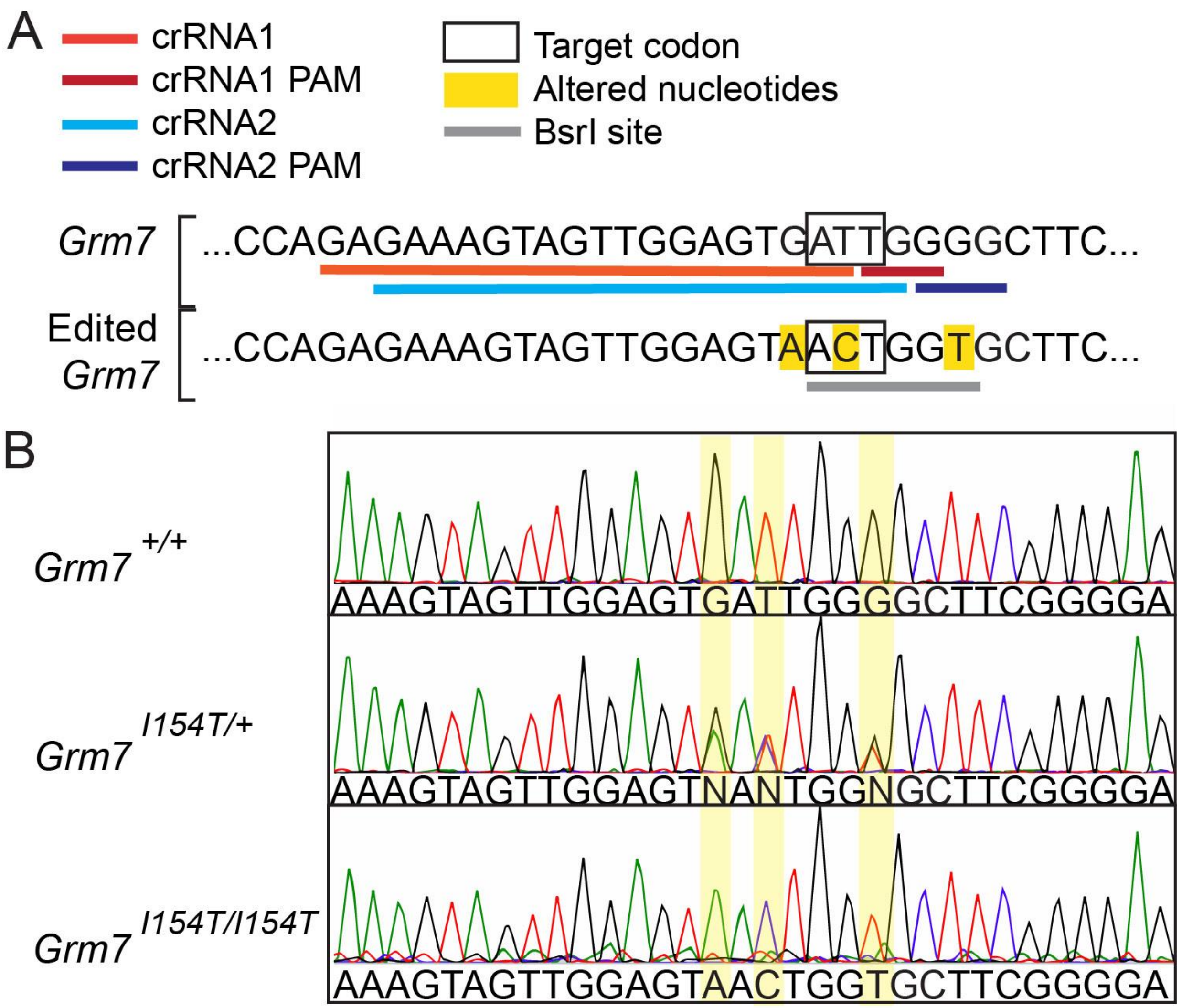

Figure S2. Generation of mGlu$_7$-I154T knock-in mouse by CRIPSR/Cas9.
A) Diagram of editing strategy targeting exon 1 of mouse *Grm7*. B) Representative Sanger sequencing results to confirm the presence of the expected single base substitutions.

Table S1. Sequences of reagents used to generate and genotype *Grm7-I154T* mice

crRNA1	GAGAAAGTAGTTGGAGTGAT
crRNA2	GAAAGTAGTTGGAGTGATTG
ssDNA repair template<br>Red text indicates bases that differ from WT *Grm7*	GCGCTTATCCAGAAGGACACCTCCGACGTGCGTTGCACCA<br>ACGGAGAGCCCCCGGTTTTCGTCAAGCCAGAGAAAGTAGT<br>TGGAGTAACTGGT<br>GCTTCGGGGAGCTCCGTCTCCATCATGGTAGCCAACATCT<br>TGAGGCTTTTCCAGgtagggggcgctccctttggggaggagcattc
Primers for PCR and sequencing	AGATCAACAGCGATCCCAAC
	CATGAAGTCCAAACCAGCTTTT

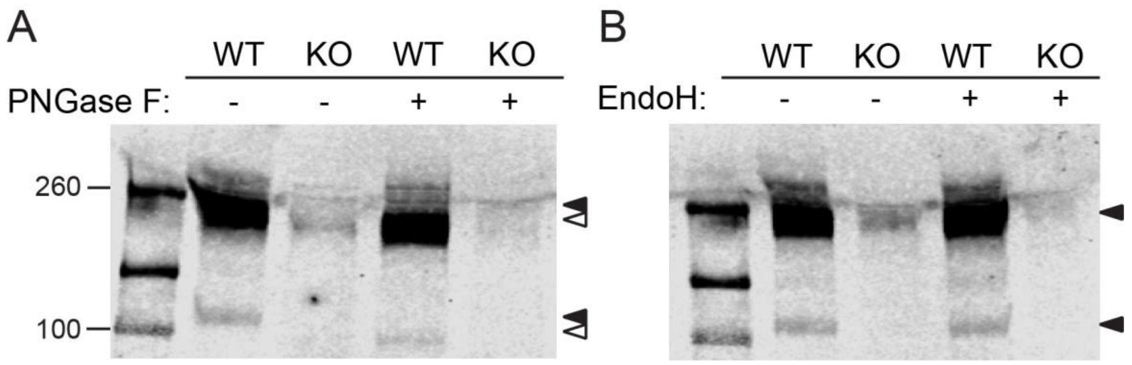

Figure S3. mGlu$_7$ detected from mouse tissue is N-glycosylated but resistant to EndoH.
Western blot of total protein lysate from mouse hippocampus in the presence or absence of A) PNGase F treatment or B) EndoH treatment. Open arrows indicate bands resulting from deglycosylation. Tissue from *Grm7*$^{-/-}$ (KO) mice was included to confirm antibody specificity.

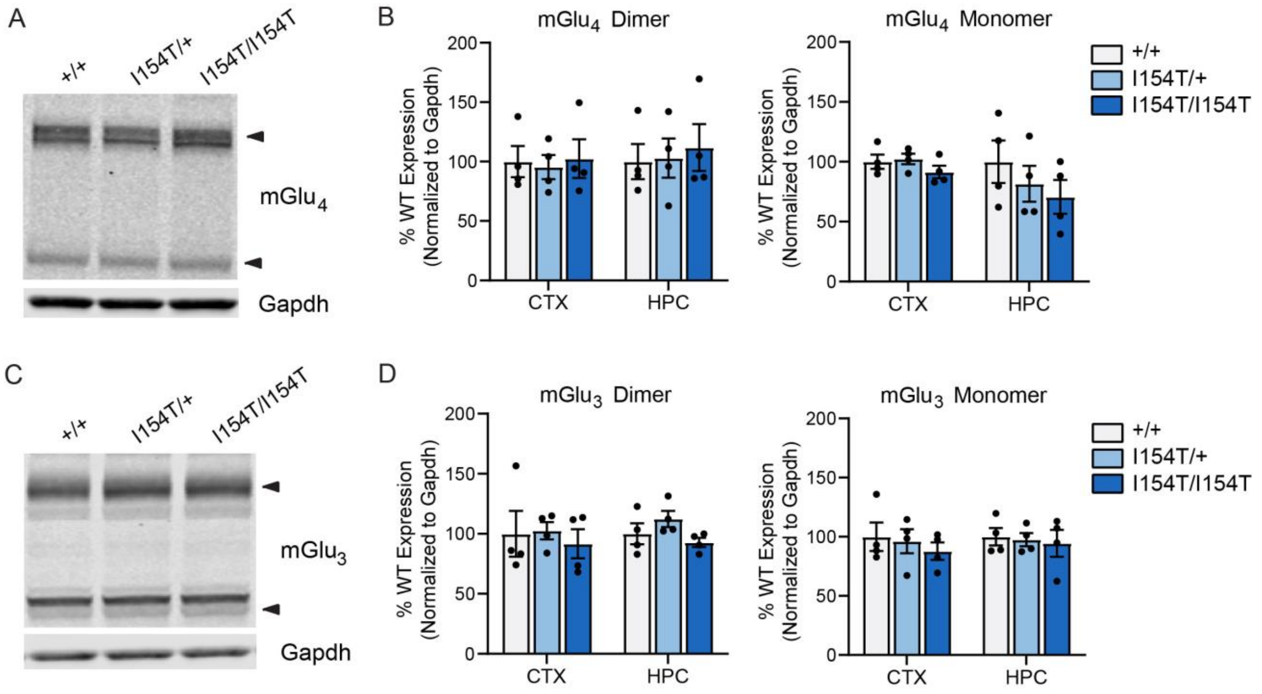

Figure S4. Expression of mGlu$_3$ and mGlu$_4$ is not altered in mGlu$_7$-I154T mice.
A) mGlu$_4$ Western blot of total protein lysate from cortex of *Grm7*$^{+/+}$ (+/+), *Grm7*$^{I154T/+}$ (I154T/+) and *Grm7*$^{I154T/I154T}$ (I154T/I154T) mice. Arrows indicate mGlu$_4$ dimer and monomer bands. B) Quantification of the mGlu$_4$ dimer band and monomer bands in the cortex (CTX) and hippocampus (HPC). C) mGlu$_3$ Western blot using the same protein samples. D) Quantification of the mGlu$_3$ dimer band and monomer bands. For all panels, N= 4 mice per genotype (2 male, 2 female).

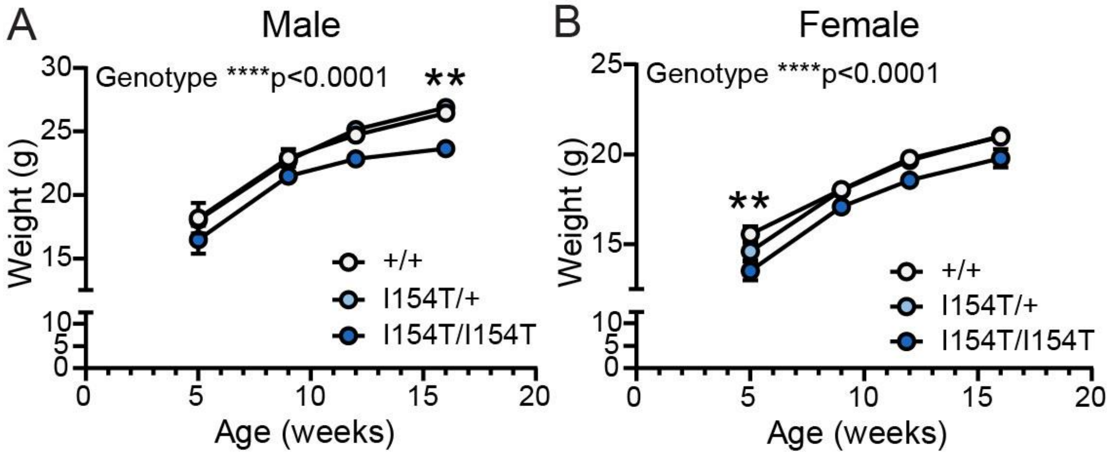

Figure S5. Mice homozygous for mGlu$_7$-I154T exhibit reduced body weight. Quantification of body weight over time of male (A) and female (B) mice. Genotypes are as follows: *Grm7*$^{+/+}$ (+/+), *Grm7*$^{I154T/+}$ (I154T/+) and *Grm7*$^{I154T/I154T}$ (I154T/I154T). For A, N = 6 +/+, 9 I154T/+, 6 I154T/I154T. For B, N = 6 +/+, 11 I154T/+, 8 I154T/I154T. Two-way ANOVA with Dunnett's comparisons to *Grm7*$^{+/+}$. **p<0.01, ****p<0.0001.

Uncropped blot images for:

A *GRM7* mutation associated with developmental delay reduces $mGlu_7$ expression and produces neurological phenotypes

Authors: Nicole M. Fisher, Aqeela AlHashim, Aditi B. Buch, Hana Badivuku, Manar M. Samman, Kelly M. Weiss, Gabriela I. Cestero G, Mark D. Does, Jerri M. Rook, Craig W. Lindsley, P. Jeffrey Conn, Rocco G. Gogliotti, Colleen M. Niswender

Figure 2C. Cell lysate (-DTT)

Anti-HA (800 Channel)

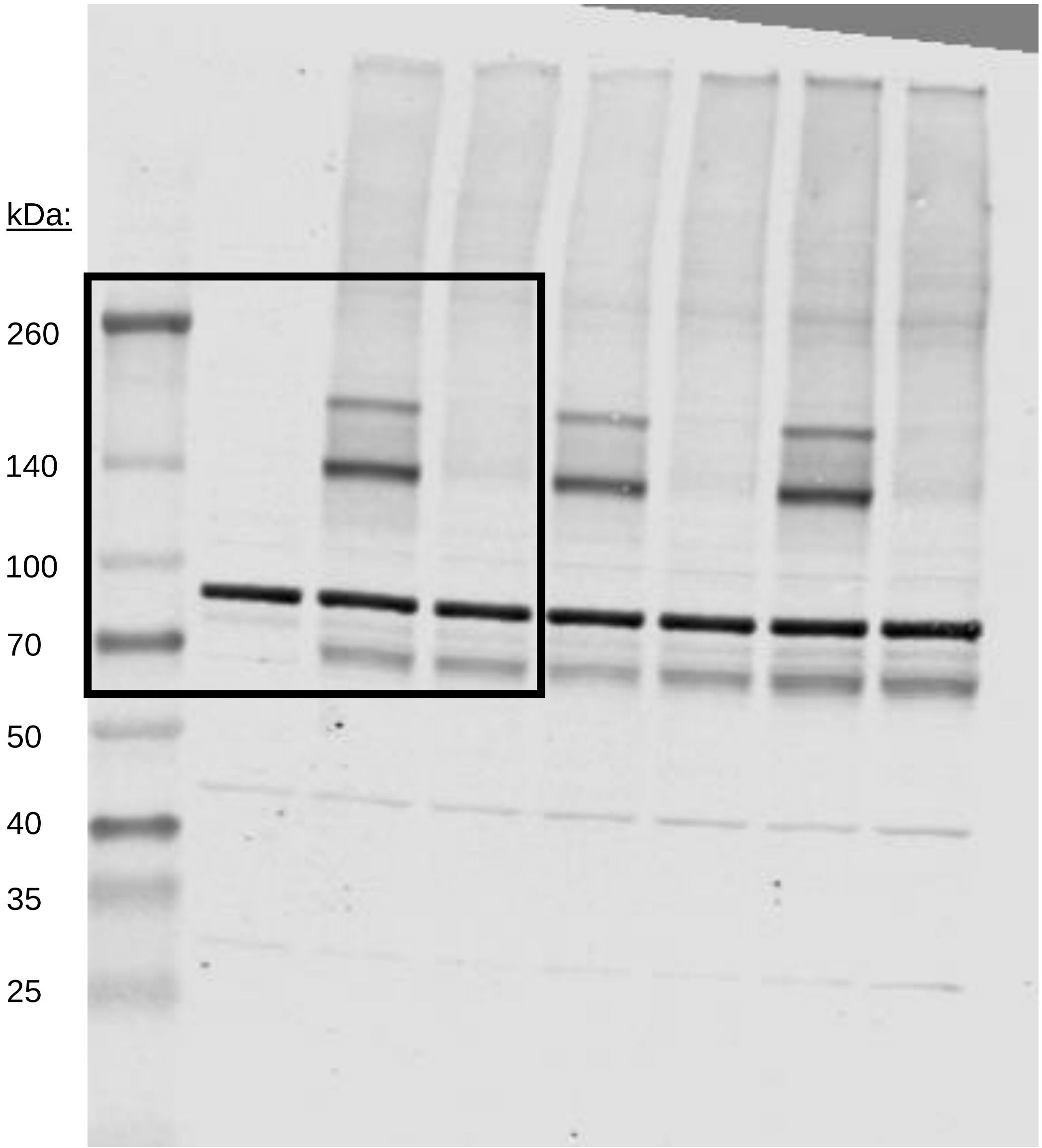

Figure 2C. Media (-DTT)

Anti-HA (800 Channel)

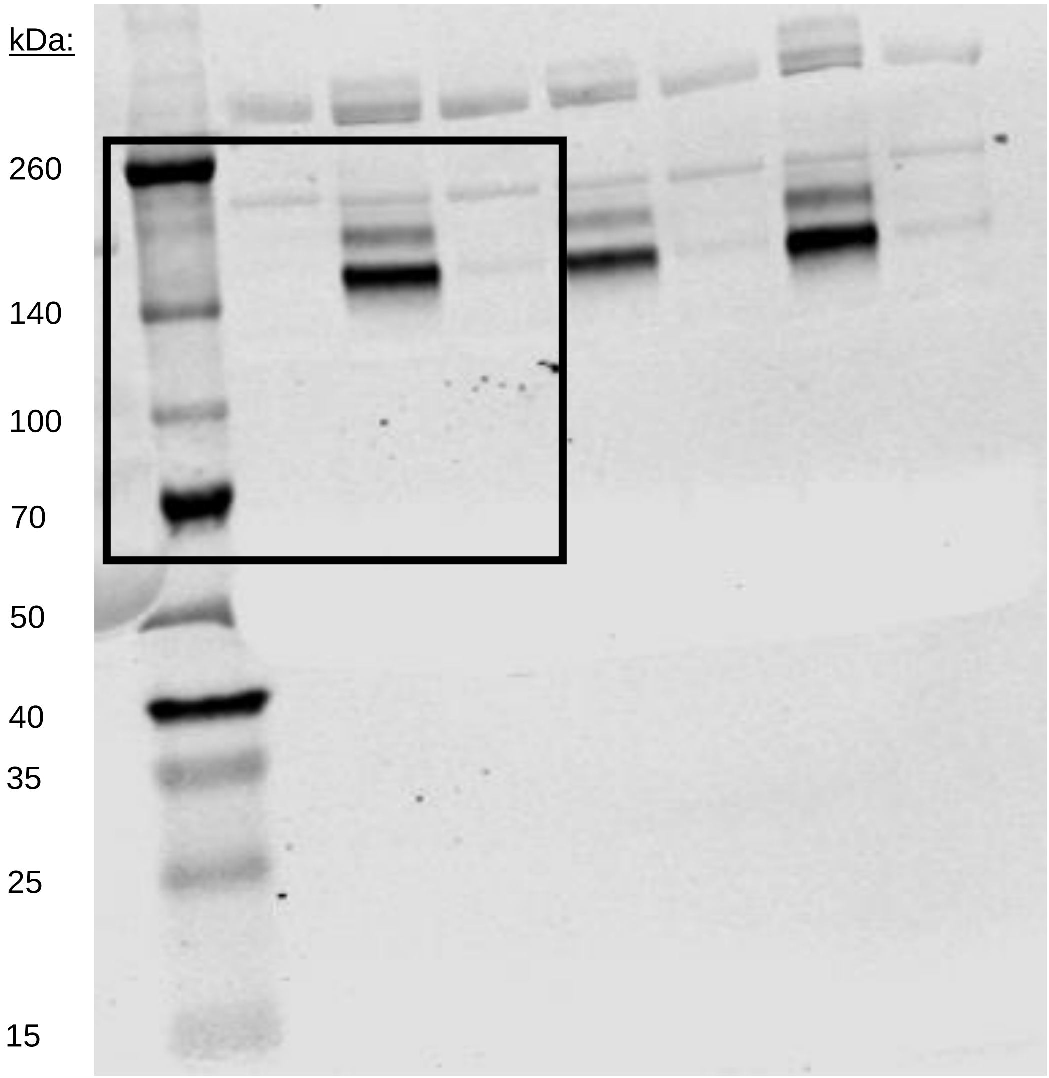

Figure 2D. Cell lysate (+DTT)

Anti-HA (800 Channel)

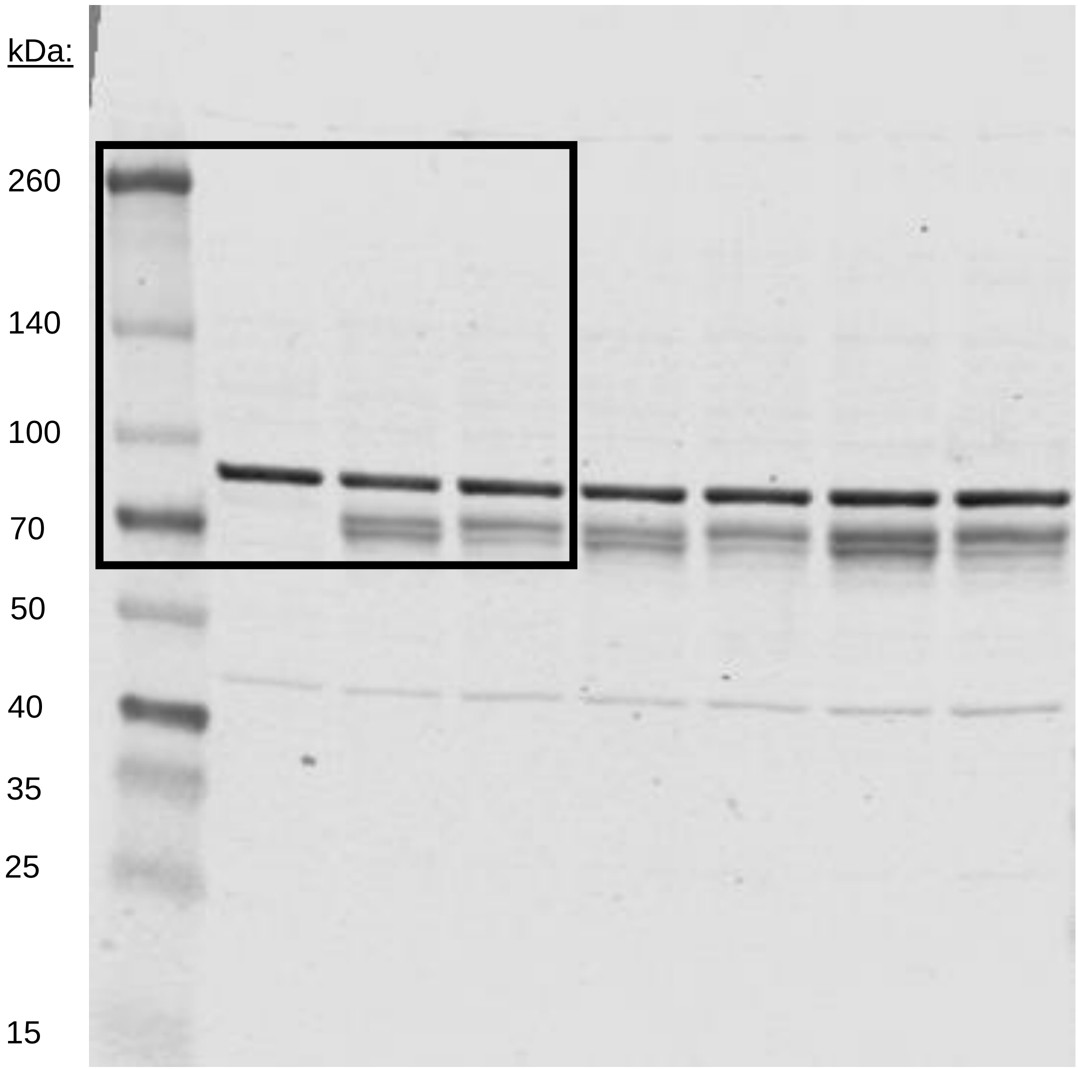

Figure 2D. Media (+DTT)

Anti-HA (800 Channel)

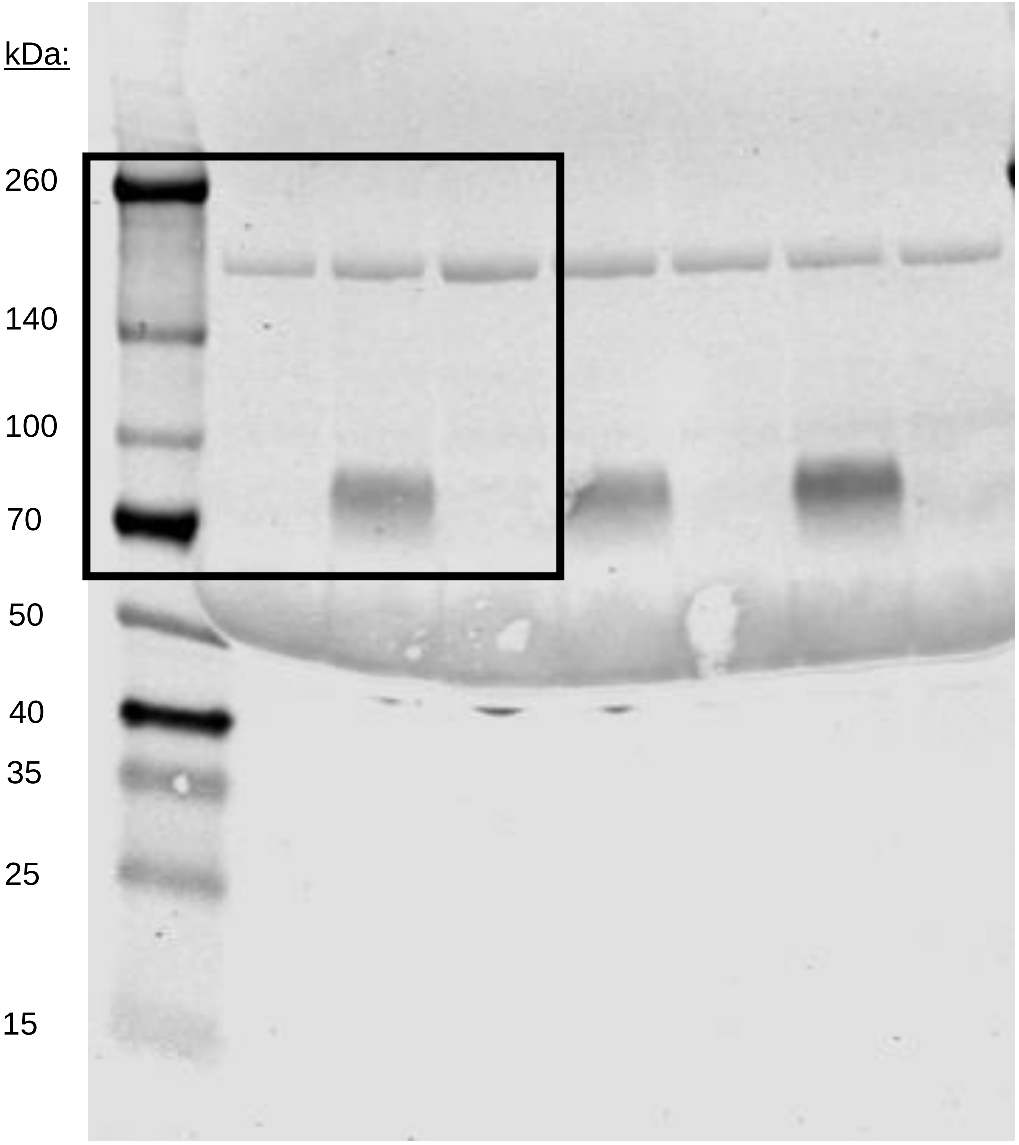

Figure 3A

Anti-HA (800 Channel)

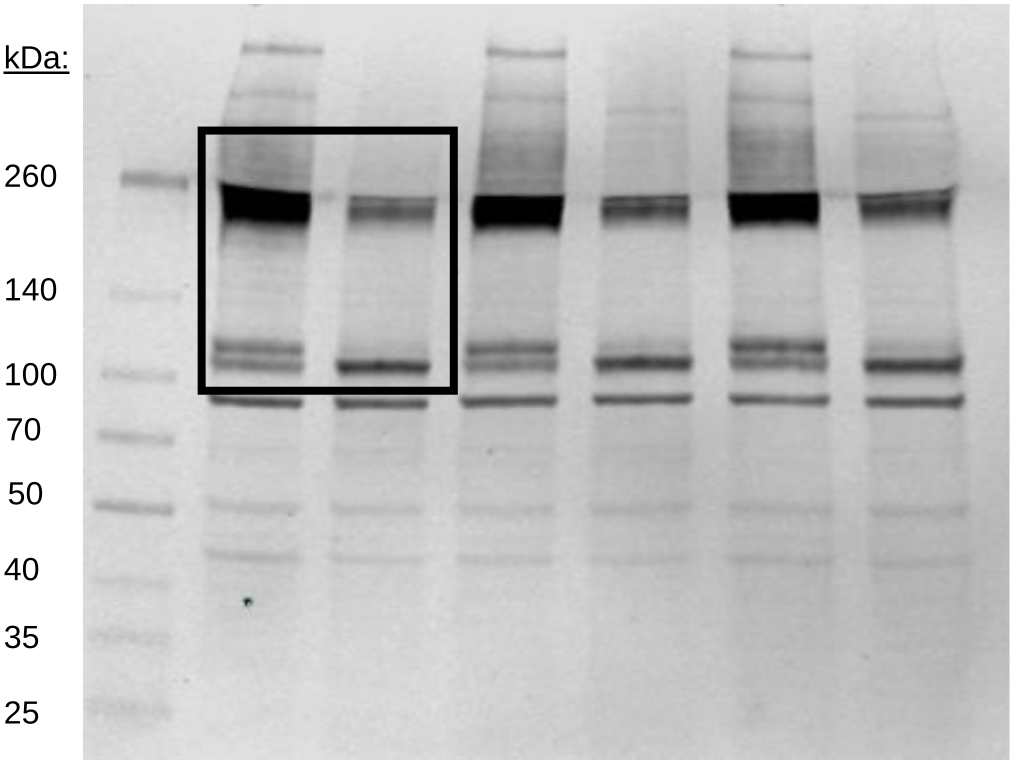

Anti-Gapdh (700 Channel)

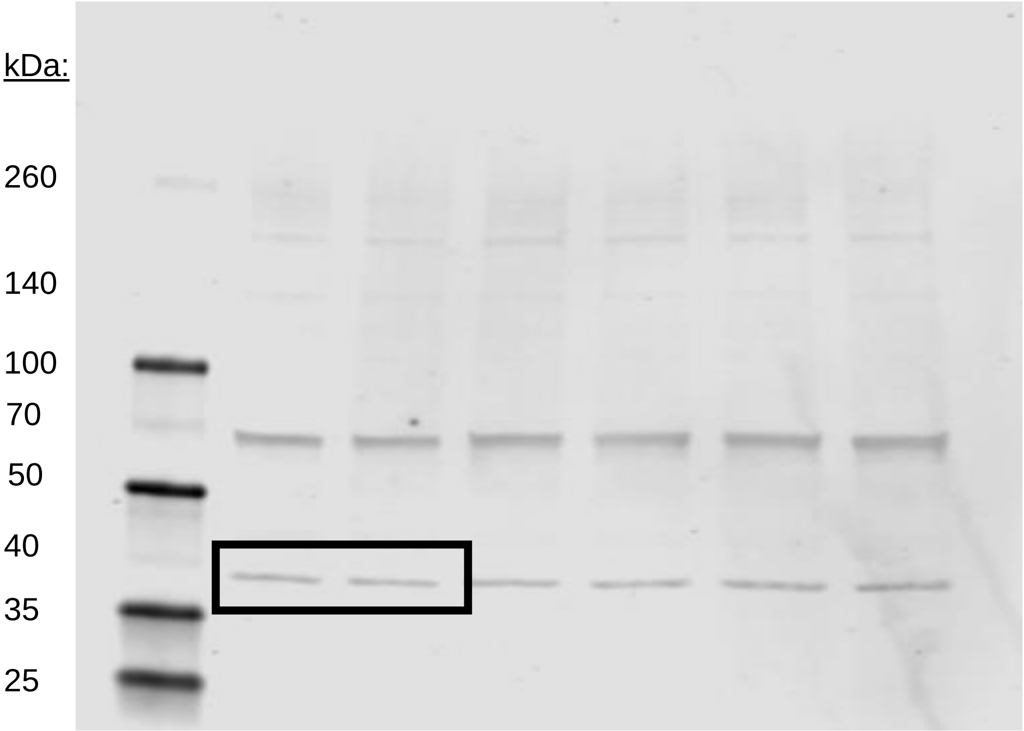

Figure 3D

Anti-mGlu$_7$ (800 Channel)

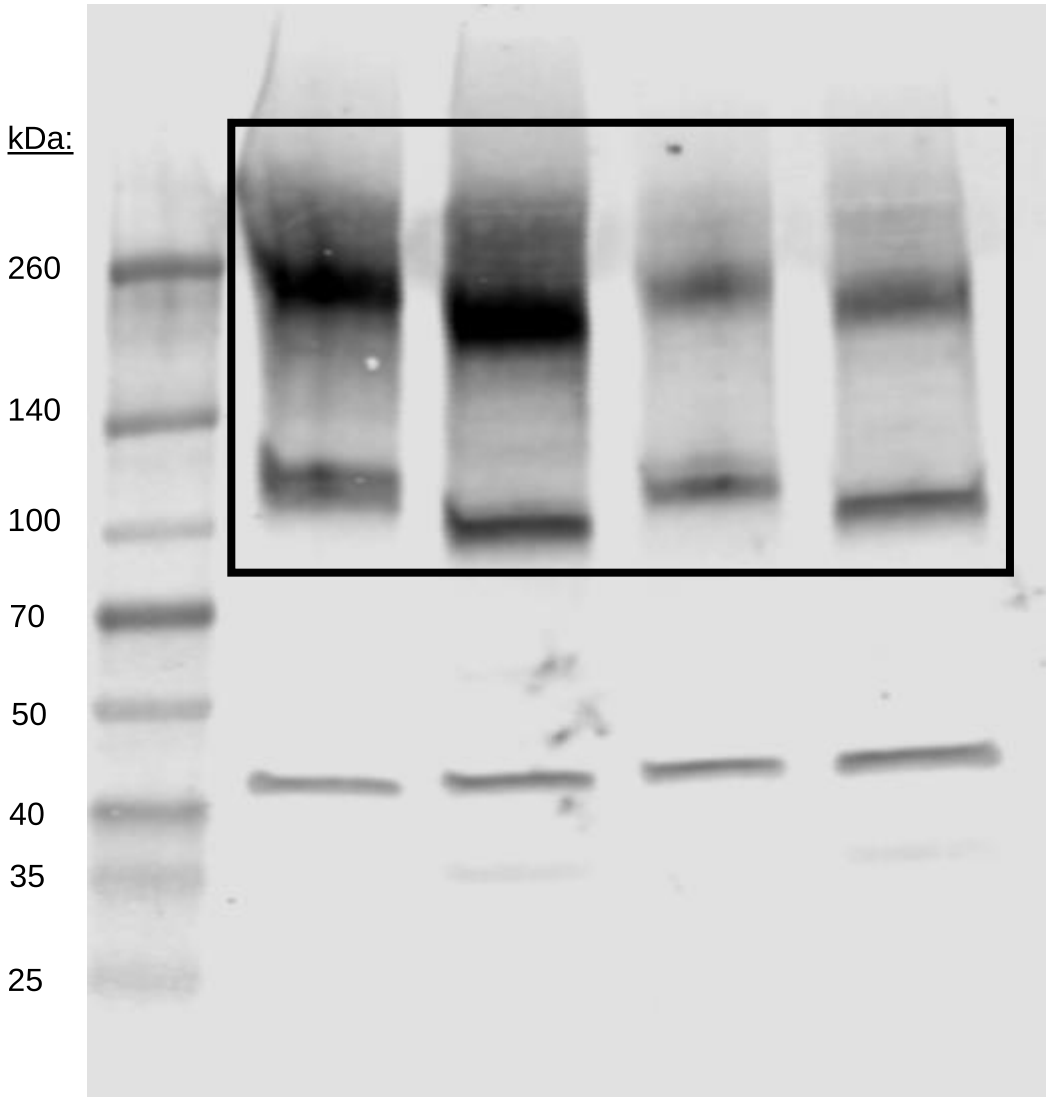

Figure 3E

Anti-mGlu$_7$ (800 Channel)

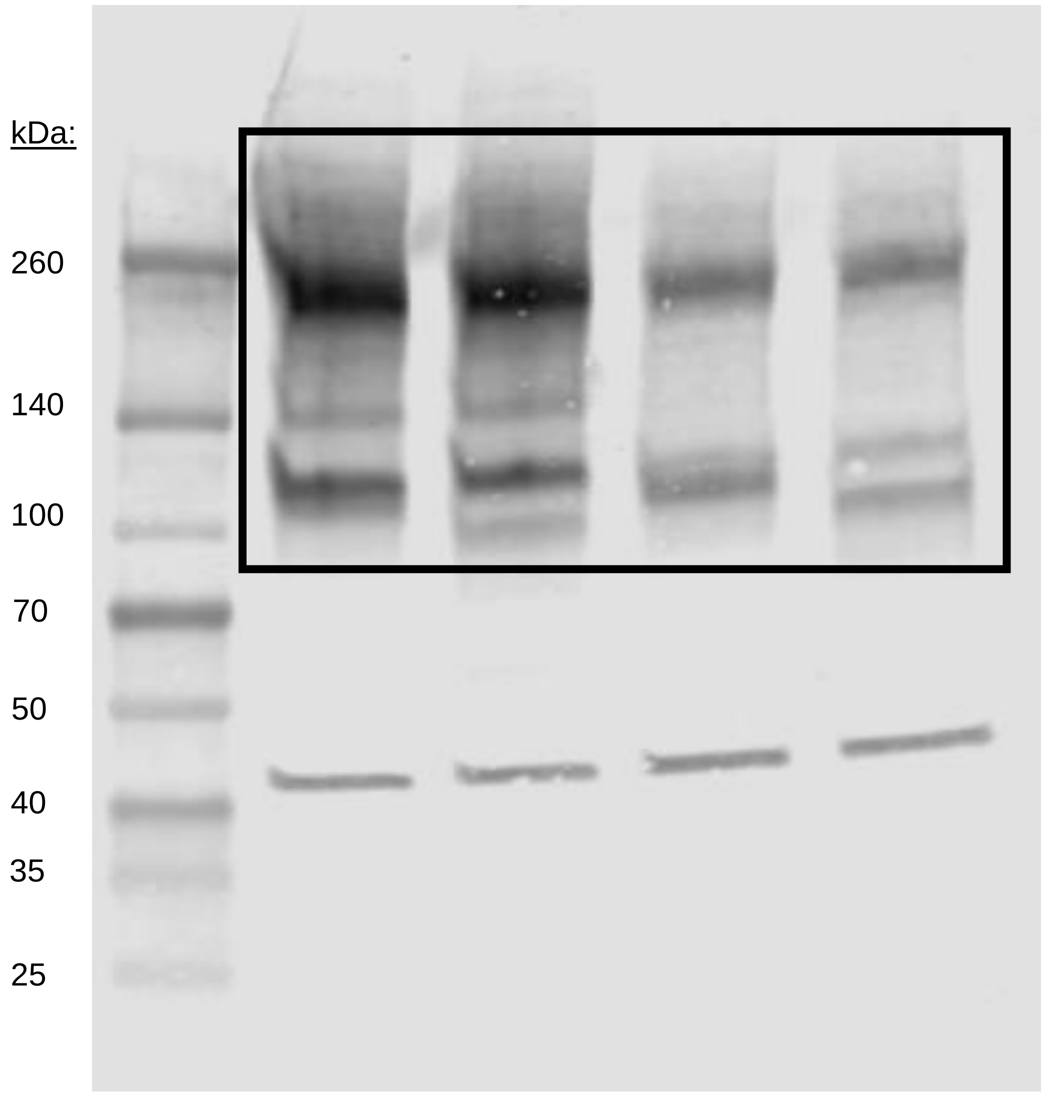

Figure 2F

Anti-HA (800 Channel)

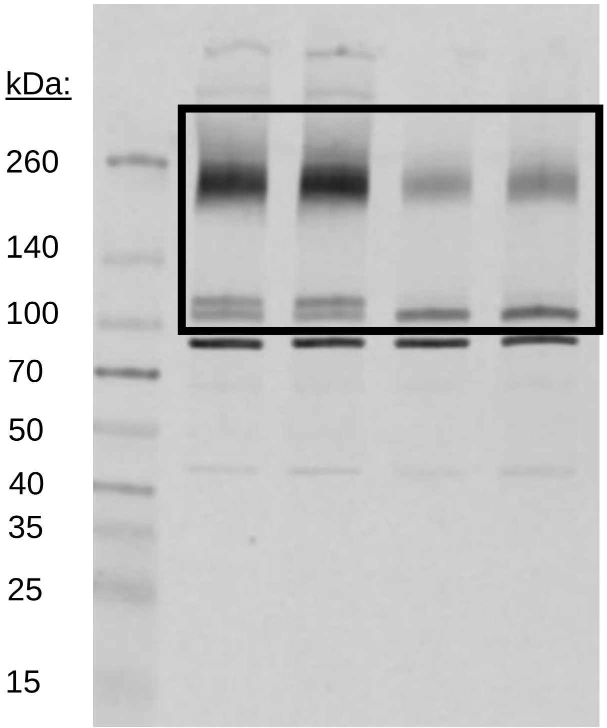

Anti-Gapdh (700 Channel)

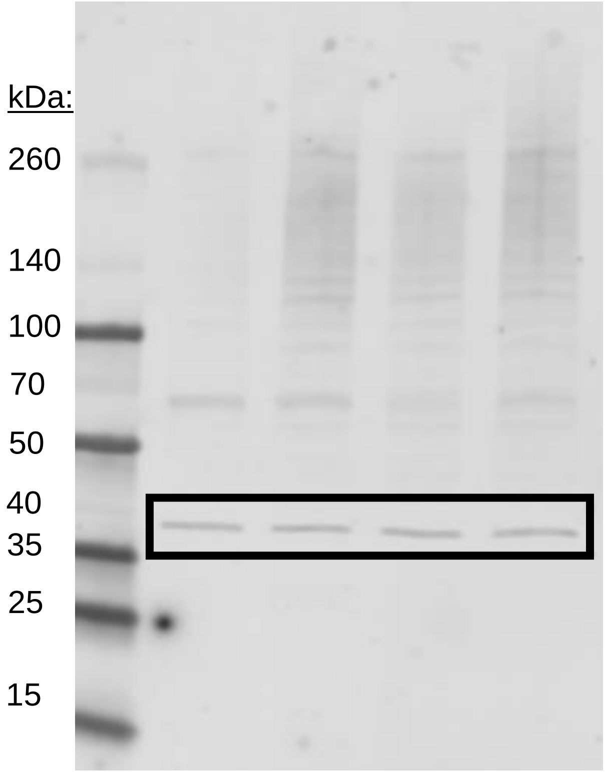

Figure 5A

Anti-mGlu7 (800 Channel)

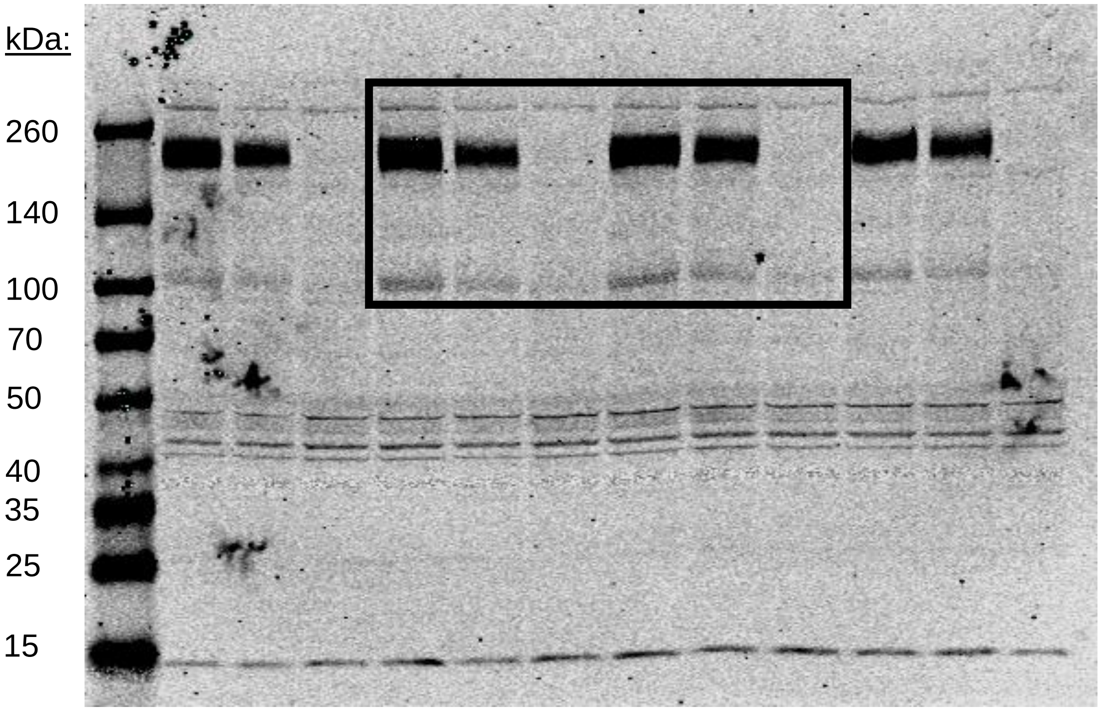

Anti-Gapdh (700 Channel)

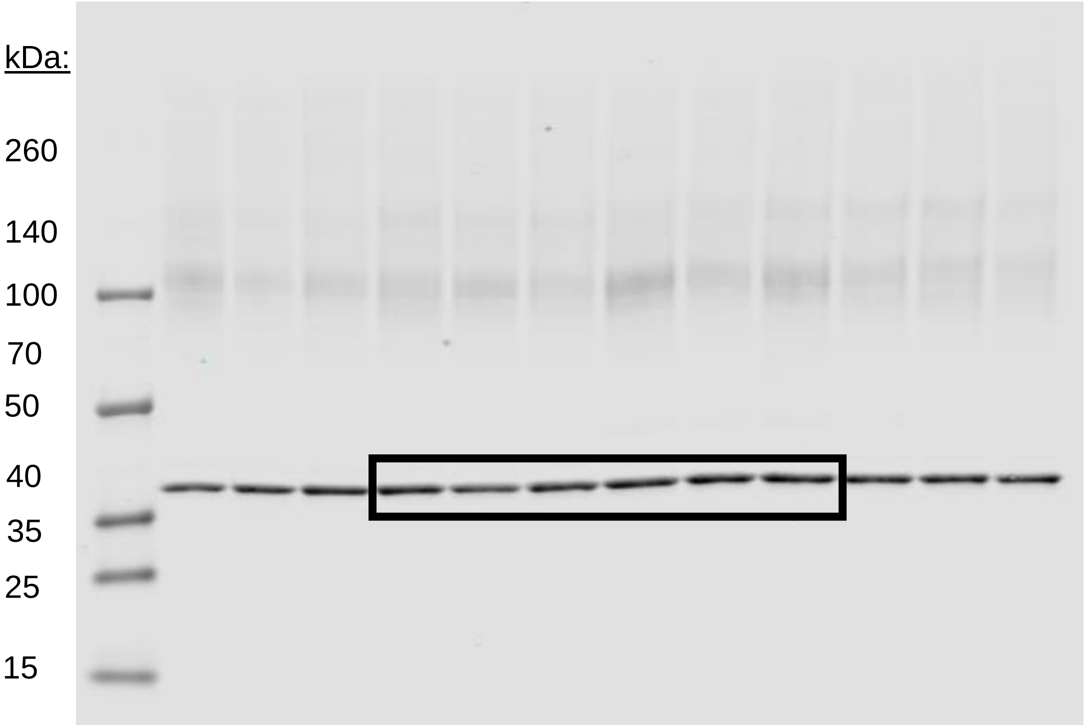

Figure S3

Anti-mGlu$_7$ (800 Channel)

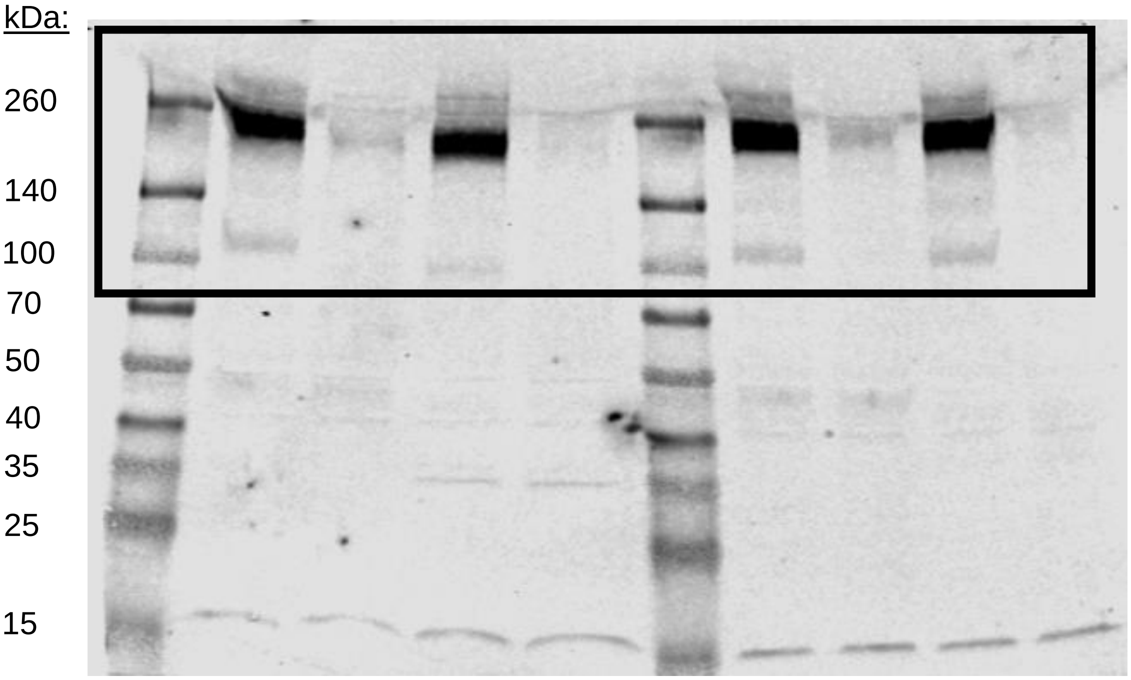

Figure S4A

Anti-mGlu4 (800 Channel)

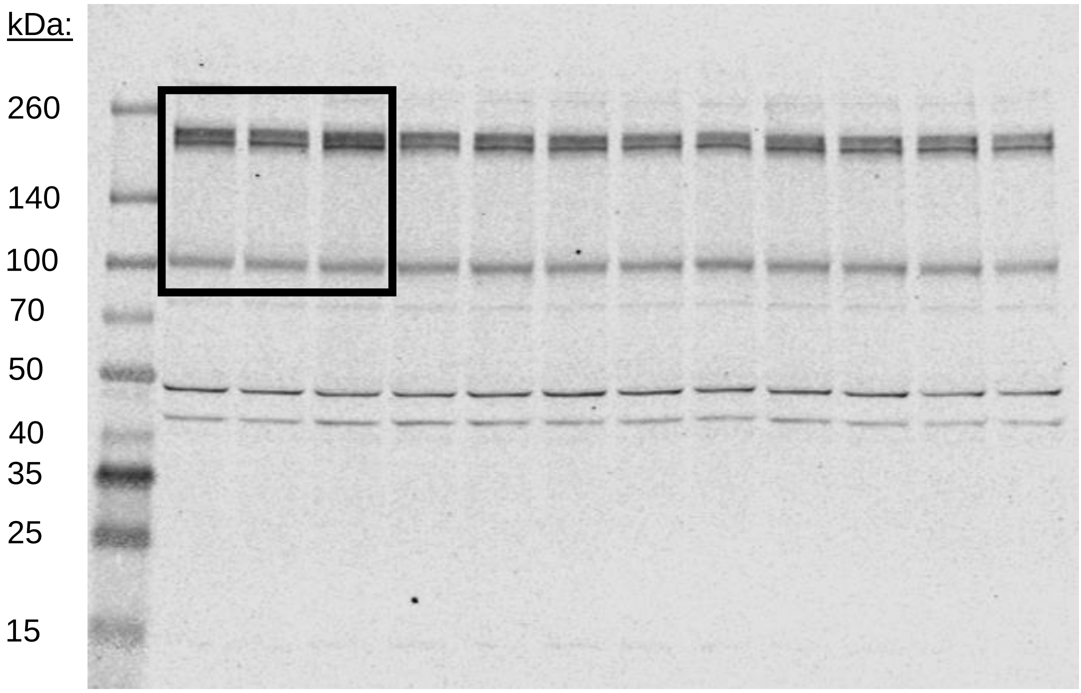

Anti-Gapdh (700 Channel)

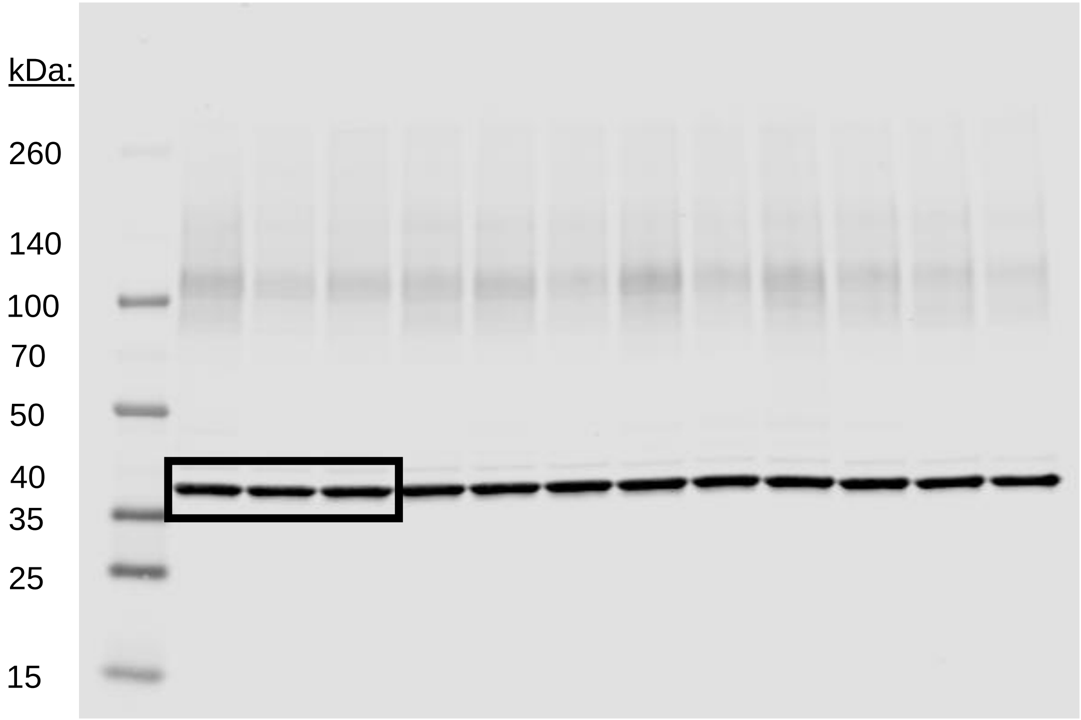

Figure S4C

Anti-mGlu3 (800 Channel)

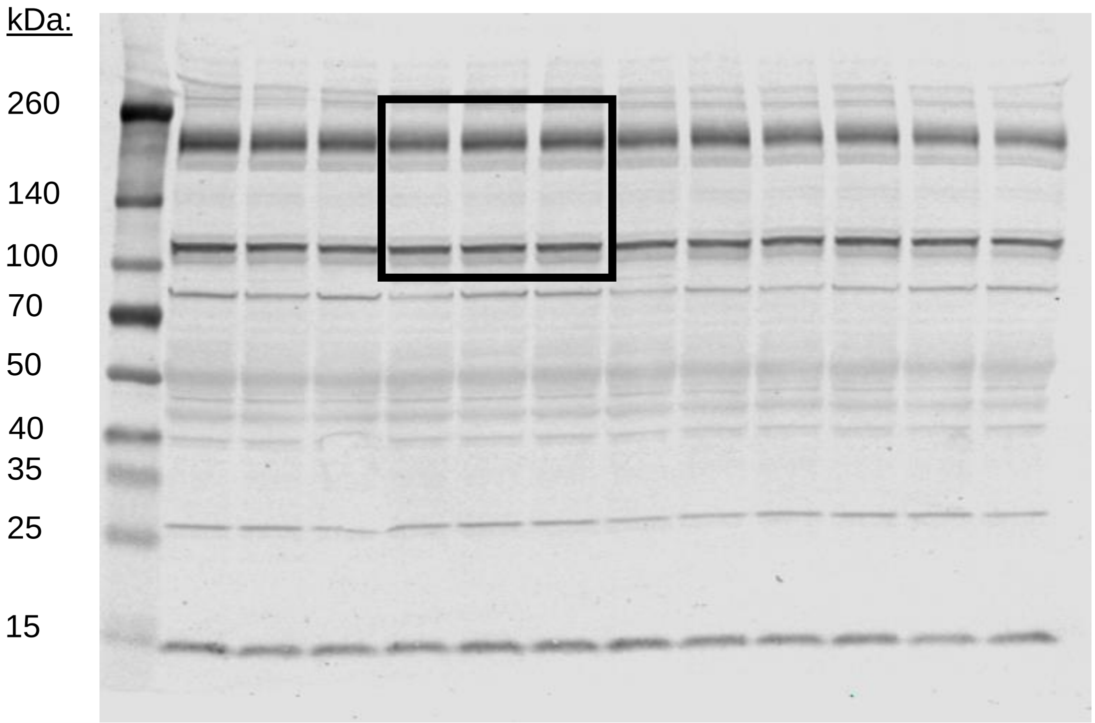

Anti-Gapdh (700 Channel)

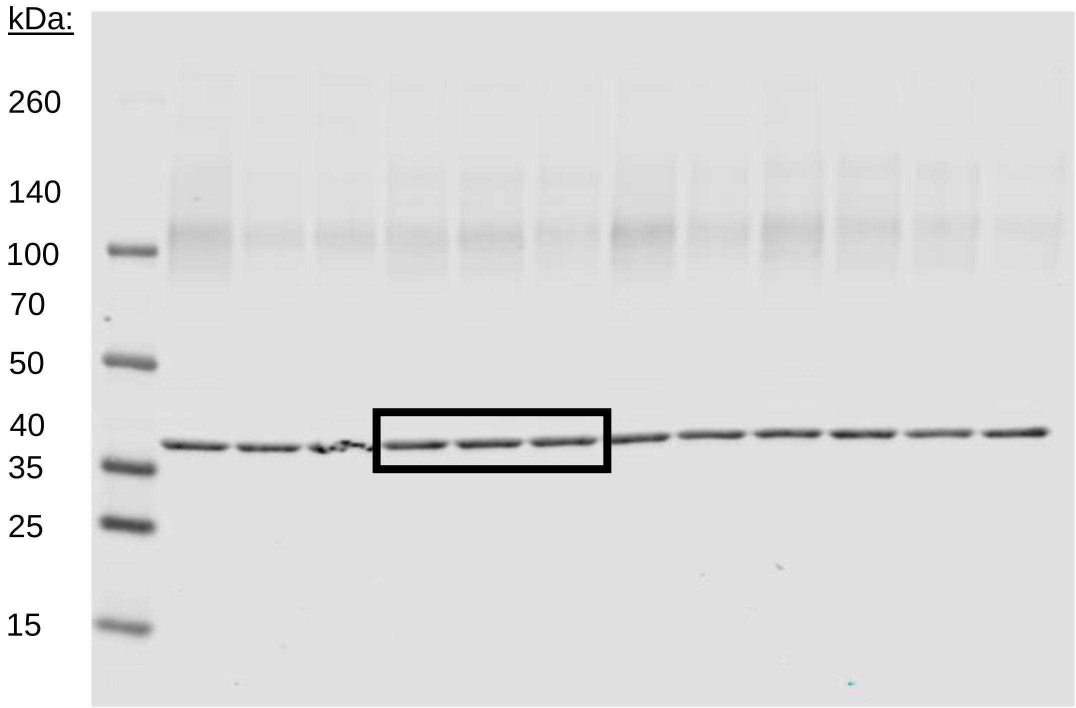

Supplement: Supplemental data [file jciinsight-6-143324-s133.pdf]
